# The architecture of Responsible Autonomy in biological and artificial systems: the Distributed Adaptive Control of volition and agency

**DOI:** 10.1098/rstb.2025.0156

**Published:** 2026-11-10

**Authors:** Paul F. M. J. Verschure

**Affiliations:** ^1^ Department of Health Psychology, Universidad Miguel Hernández de Elche, Elche, Spain; ^2^ Instituto de Neurociencias (CSIC-UMH), Universidad Miguel Hernández–Consejo Superior de Investigaciones Científicas, Sant Joan d'Alacant, Alicante, Spain

**Keywords:** free will, volition, agency, Responsible Autonomy, Distributed Adaptive Control (DAC), ESRA, allostatic control, cognitive architecture, AI governance, CESP cascade

## Abstract

This paper proposes the Distributed Adaptive Control theory of volition and agency (DACva), which parses biological and artificial free will into six capacities that close the agency gap between *a reflex and an act*: active decoupling, endogenous drive, leeway, normative self-governance, diachronic integrity and experiential ownership. Together they constitute Responsible Autonomy. The DACva architecture spans five layers, somatic to reflective, coordinated by a decoupled model-predictive controller, the compression–expansion–selection–projection (CESP) cascade. It constructs, rather than presupposes, the causal models counterfactual control requires, under the evolutionary pressure of survival in social worlds pervaded by hidden intentional states. The computational principles and neurobiological substrate of DACva are specified in a 12-equation formalism, with an assay and a refutation condition for each capacity. The formalism closes on one equation, the CESP cascade, and yields an explicit definition of freedom: the degree to which an act is the committed trajectory of the agent's own cascade, read on six conditions, one per capacity. From this follows the Evaluation Suite for Responsible Autonomy (ESRA), a perturbational test of architectural competence rather than mimicry, proposed to replace the imitation game; ESRA-S assesses the safety constraints a deployer of AI must impose. DACva argues that the architecture of Responsible Autonomy is the architecture of mind.

This article is part of the discussion meeting issue ‘From being to identity: analysis and synthesis of the self’.

*‘That thing is called free which exists from the necessity of its nature alone, and is determined to act by itself alone.’*

Spinoza, *Ethics*, I, Def. 7 (1677/1985)

## 1. Introduction: the synthetic paradigm of Responsible Autonomy

In the summer of 1943, after the Prosper resistance network was dismantled, Noor Inayat Khan, a Special Operations Executive wireless operator in occupied Paris, declined London's offer of extraction. Remaining meant working alone and evading capture while transmitting the network's last radio link, a choice that cost Noor her life. She was shot at Dachau, where a witness reported her final word as ‘Liberté’ [1]. How should we understand Noor's sacrifice? Her decisions were neither reflex nor compulsion, but a cognitive and emotional feat: representing alternatives, evaluating consequences, holding a course across time and acting from values treated as one's own, against mortal threat. This is the phenomenon that folk psychology labels ‘free will’, the presumed capacity that makes an agent a fit target for blame, credit and respect, and so the foundation of moral accountability. The pursuit of free will as an ontological absolute has left the sciences of mind with a choice between an uncaused spark and a deterministic reductionism. Reducing Noor's sacrifice to an epiphenomenon, an illusion or the ‘bad luck’ of a biological automaton caught in a causal crossfire leaves the difference between her act and a reflex, the agency gap, unexplained and unvalued. This gap is not a discovery about agents but a consequence of a dogma, the exactitude fallacy: the ontology and epistemology of physics applied to mind and brain without adaptation. The gap admits two moves only: to save the dogma, one must eliminate the agent, and to save the agent, one must eliminate the dogma. This paper takes the second.

The dispute has so far been conducted from the armchair. The rise of advanced artificial intelligence (AI) cannot wait for it to stand on its priors and opinions and advise society to govern a phantom (Part 1 [2]). The way out is to bridge the agency schism, between the dominant philosophical and neuropsychological consensus of illusionism [3–5], the denialism of prevailing ethical analyses [6,7], the industry's persistent existential-risk narrative [8] and the societal need for comprehension and regulation of AI [9–11]. Governance, confronting artificial agency, bypasses the academic dispute entirely: the regulatory frameworks engage none of the tradition's canonical works and define autonomy operationally, reaching unaided for a naïve ethics of human exceptionalism. These endeavours are stuck for the same reason: neither can say what an agent is. Regulators must decide whether an artefact is a fit subject of accountability, and a tradition that denies the agent or cannot build one cannot tell them. The one system known to close the agency gap is the brain. This paper therefore advances a theory of brain function in order to understand and govern a putative alien volition constructed by humans. The dependency runs both ways: the natural case supplies the mechanism; the artificial case supplies the test, by building that mechanism and facilitating perturbation and access that the brain cannot. Stated as an architecture rather than as biology, the mechanism can be measured in any substrate. Part 1 read the contemporary free-will tradition as a degenerating research programme [12] and distilled from its failure modes six positive capacities. Based on this, I advance a progressive problemshift from governing a ghost to benchmarking a mechanism by dissolving the metaphysical question into a pragmatic one and parsing the phenomenology of free will into the structure and function of the six capacities of Responsible Autonomy that close the agency gap (table 1). This problemshift is consistent with a return to pragmatism in the philosophy of biology and neuroscience, mediated through models judged by what they let one build, intervene on and predict [13,14], capturing Giambattista Vico's verum ipsum factum [15]: we can understand what we construct. In this synthetic epistemology, a capacity is understood by constructing a model that exhibits it, satisfying convergent constraints from concepts, anatomy, physiology and behaviour, and falsifiable through targeted perturbation [16–18]. This is the move from metaphysics to engineering: the agency gap becomes a tractable control problem, and free will, so parsed, is naturalized as Responsible Autonomy. This step rests on one equation: (*Ξ*,ξ*, *a*) = CESP(*Ψ*, *u*) (equation (2.3)) where an act is the output of the agent's own cascade over its state *Ψ* and its input *u*. *Ξ* is the ensemble of candidate trajectories the cascade generates, and ξ* is the one it commits to. Each capacity is a term or a gate of that cascade. Free will is not a further ingredient but a property of how the equation is closed, and the paper ends by defining freedom in these terms. Here Sellars's space of reasons [19] is neither reduced to the space of causes nor an irreducible given, but emergent and real: the Distributed Adaptive Control theory of volition and agency (DACva) neither eliminates norms nor posits them sui generis; norms gate choice and action through dedicated mechanisms (capacities III–V). Their justification is left to the communal realm, the sociostasis of the socially situated agent (capacities I, II and IV). DACva enters not as a fresh conjecture but as the current state of a progressive research programme, whose core generates novel predictions and is revised against them across over 370 derived studies spanning computational models, animal and human data, autonomous robots, and clinical and educational applications [20].

**Table 1.** The six capacities of Responsible Autonomy, the top-level control requirements that close the agency gap, with the principal DACva metric that operationalizes each. (Their philosophical lineages, evolutionary drivers and failure modes are derived in Part 1 [2]; key concepts are defined in the capacity sections and in the supplementary material A (SA).)

I. Active decoupling: self is actively decoupled from non-self; μ, see equation (2.4*a*)
decoupling is hierarchically controlled relative to viability; trust in the decoupled model rises with its reliability for the valued goal, stays bounded above a non-negative floor, falls faster than it recovers and combines anticipation, recall and authorship discrepancies
II. Endogenous drive: self is moved by its own needs, not by supplied reward; $\hat{\bar{A}}$, *Ĉ*, see equation (2.5)
viability grounds action through allostasis, segregating appetitive and aversive value; net drive is their difference, conflict their sign-invariant co-activation, which raises the evidence needed to commit; the aggregates are hierarchically organized and score a candidate's predicted effect on need
III. Leeway: self constructs more than one genuine path; ℒ , see equation (2.7)
leeway increases with the breadth of viable alternatives, vanishes when generation collapses to one or the dynamics become unstructured, stays bounded by the agent's self-anchored causal model and history, counts only alternatives made available to selection and depends non-monotonically on the substrate's dynamical regime
IV. Normative self-governance: self is bound by norms it holds as its own; β, gate *g*, see equations (2.8) and (2.9)
physical and social value combine on a common scale under a bounded convex weight with positive reserve, so viability is never erased; prohibition is a property of the acquired norm, not intrinsic to the agent; commitment operates on a candidate trajectory that is coherent, model-supported and decisive relative to conflict; withholding is causally distinct from choosing inaction
V. Diachronic integrity: self stays one and the same across time; 𝒯, see equation (2.10)
integrity jointly rewards depth, concentration and coherence of mental traversal, contracting the ensemble onto segments supported by evidence, succession and value
VI. Experiential ownership: self owns the acts it authors; 𝒜 = $\bar{\rho }$, χ, see equations (2.11) and (2.12)
Ownership holds for covert/unreleased and overt trajectories in agent-defined episodes, is assigned by default and reduced by intention and control error and is independent of outcome success; efficacy is measured intrinsically against the agent's realized policy and active self-regulation, not injected value

In the DAC framework, Responsible Autonomy is an evolutionary control response to the transition from physical survival in How, Why, What, Where and When (H4W) to social environments pervaded by the hidden intentional states of others, the Who in H5W [21,22]. This transition creates two computational bounds. Deciding whether there exists a joint policy that attains a specified reward in a finite-horizon decentralized partially observable Markov decision process is NEXP-complete with as few as two agents [23], so entry into social worlds cannot be bought with computation alone. Evolution replaced the problem rather than solving it: the agent satisfices [24], substituting a good-enough own policy for an optimal joint one, bounded by what its own viability can tolerate. The second bound is individual: unrestricted recursive modelling of others produces an unbounded hierarchy of nested beliefs, so modelling depth must be controlled [25]. The Intentionality Prior is the bounded inductive response to both [22]: it segments observed dynamics through a restricted set of inferred agents, goals, beliefs and intentions [26–28], and the depth bound itself is derived, not stipulated (diachronic integrity, capacity V). DACva defines Responsible Autonomy as an integrated agent-centric control construct for H5W worlds in which the agent is simultaneously a fit subject of accountability practices and an active participant in them. It is predicated on this substitution: an agent that acts socially at all is one that has replaced the intractable problem with a bounded one it can afford.

This paper takes the next step: a theory of the natural case, DACva, built to be tested on the artificial one. The six capacities are requirements of Responsible Autonomy; what is needed is the mechanism that meets them so they can be perturbed and measured. The account of the brain developed here is architectural: canonical DAC (§2). DACva translates each capacity into a control-architectural commitment, states a candidate mathematical realization and names the neural substrate hypothesized to carry it (capacities I–VI). Once the mechanism is specified, a test follows: the Evaluation Suite for Responsible Autonomy (ESRA) assesses biological and artificial systems on the six capacities under perturbation. Each capacity has a refutation condition, and a causal criterion separates a realized capacity from its mimicry. The deployment constraints asked of an artefact are scored apart from the evidence for its autonomy (§3, Measuring Responsible Autonomy). The Outlook states where DACva is being built and applied; the Discussion answers the main objections, states its limits and compares it with observer-side accounts and takes stock of what has been achieved. DACva is a candidate theory assessed against the test it makes possible.

## 2. Capacities of Responsible Autonomy

The six capacities of Responsible Autonomy are a term or gate of the DACva CESP cascade. Each is set out on three levels: (i) the architectural requirement that DACva claims must hold; (ii) a candidate mathematical realization of that requirement grounded in the DAC research programme; and (iii) an outline of the proposed biological implementation, given as the substrate constraints first and then, for each element of the formalism, the system hypothesized to implement it. Lastly, the ESRA test (§3) states the interventions that would falsify each mapping and the internal quantity each intervention must move before behaviour changes. Notation and conventions are given in the supplementary material, SA.

### (a) The Distributed Adaptive Control theory of volition and agency architecture

DACva builds on the canonical DAC formulation of agent-centric multilayered control (figure 1), from the reactive–adaptive architecture through sequential episodic control, anticipation and allostatic regulation to its current benchmarks [18,21,28–37], hereafter canonical DAC. DAC is not one architecture among many [38]; its taxonomy of layers and columns locates all extant proposals such as the subsumption, SOAR, ACT-R, real-time control architecture, and the current deep reinforcement learning and foundation-model systems, as partial instantiations of one layered control system [39,40].

**Figure 1.**
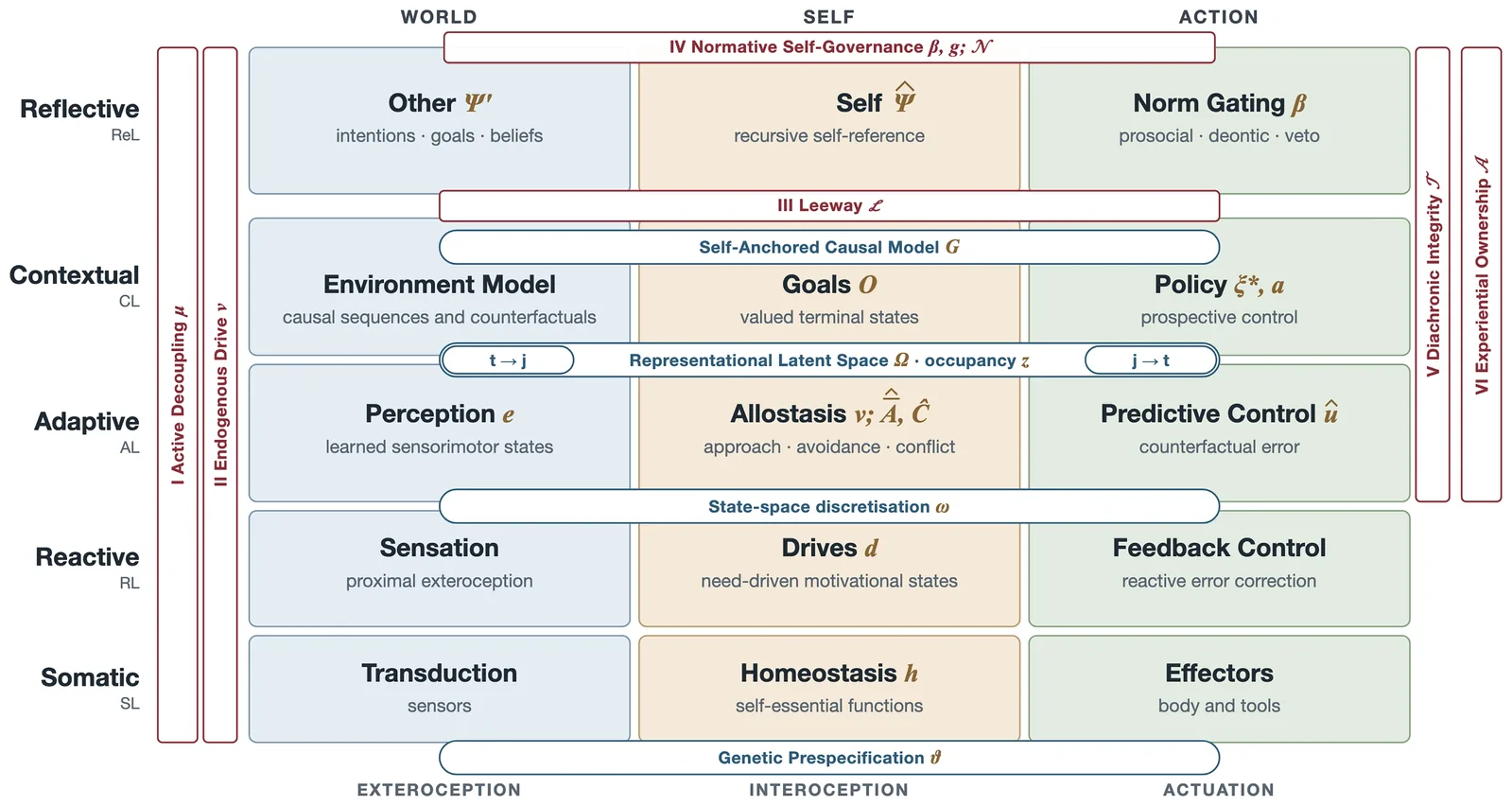
The DACva architecture. Five hierarchical layers (somatic, reactive, adaptive, contextual and reflective) intersect three functional columns processing states of world, self and action along the exteroceptive, interoceptive and actuation modalities. Each cell names the function constituted at that intersection. Four horizontal bands trace the bottom-up construction of genetic prespecification ϑ at the somatic level, the discretization of the continuous sensorimotor space into conjunctive state couplets ω at the reactive/adaptive border, their compression into the representational latent space Ω at the adaptive/contextual border and the self-anchored causal model *G*, whose nodes are formed from Ω across the contextual layer and whose dynamic-memory occupancy *z* is the transient state the compression–expansion–selection–projection (CESP) cascade evolves. In the simplest anatomical reading, Ω is hippocampal and *G* is neocortical (capacity III, leeway). The six capacities of Responsible Autonomy with their metrics are drawn as bars, placed where they are constituted. The two inserts in the Ω band mark conversions between real time *t* and mental order *j*. See text for further explanation.

The somatic and reactive layers of DAC (SL and RL) establish foundational physical autonomy, mapping exteroceptive, *e*, and interoceptive, *n*, input to action, *a*, via homoeostatic drive regulation, *h* and *d*. The adaptive layer (AL) learns the perception–action state space through predictive, counterfactual learning implemented as a re-entrant expectancy loop driven by reconstruction error. It learns the first of two levels of causality, world causality: which stimulus predicts which, relative to the agent's internal state, as in classical conditioning. Perception and action then follow the predicted rather than the present stimulus [18,41,42]. These states are compressed into sequences of conjunctive couplets of perception, value and action, *ω* = (*e, v, a*), constructing a representational latent space *Ω* [18,30,43]. The contextual layer (CL) learns the second level, agent–world causality, operantly from the agent's own actions. It builds the self-anchored causal model *G* = (*V_G_*, *E*, *ℱ*). Its node set *V_G_* holds the segments, each a stored couplet *ω* with its place in a sequence and the goal and valence marker of that sequence, acquired as sequences of episodic memory states during active engagement. Transitions are defined by directed edges *E* and content-addressed retrieval dynamics *ℱ*, defining the occupancy, *z,* of DAC's dynamic memory (DM). *Ω* holds recall frames over the segments of *G*, the two forming complementary memory systems, with consolidation at the goal state (supplementary material SA, S6.1).

*G* is more than a world model and is self-anchored. At the H5W level, it must include the hidden intentional states of others and the self and the norms among them. DACva anchors these to the agent's own structure and viability, its self-frame *s*. *G* therefore ranges over self, others and norms as well as the physical environment in the agent's frame of reference. It is closer to the small-scale world model Craik proposed, which the agent carries of both external reality and its own possible actions [44], than to the world models of contemporary machine learning. Where CL delivers policy decoupling, the reflective layer (ReL) facilitates social decoupling through metacognitive processing for self–other modelling and the extraction and application of social norms [21,22,28,45]. In compressed form, the state of an agent, *Ψ*, is defined by separating fast control variables, *x*, from a slower structural state, *W*. This structure includes genetically controlled parameters, *ϑ*, of which a constitutional subset *ϑ*_0_ is held fixed over the lifetime to define temperament, while the remainder is open to neuromodulatory and endocrine control on timescales between *x* and *W* (see the supplementary material, SA). The split follows the separation in canonical DAC of state variables from structural state and from the genetically fixed values of its SL and RL (supplementary material SA, table S7.1):

(2.1) Ψ=(x,W),x=(h,d,v,z,μ),W=(Ω,G,ϑ),

with *h* the need state, *d* the drives, *v* the value state, *z* the occupancy of dynamic memory and *μ* the agent's trust in *G*. The dynamics of the agent evolve on two clocks: physical time *t* and decoupled mental order *j*. The agent state advances based on extero- and interoception, changing its memory systems and allostatic state, the coupled loop of the RL, AL and CL of canonical DAC (supplementary material SA, table S7.1):

(2.2) $$\Psi_{t+1}=D\left(\Psi_{t},u_{t}\right),u_{t}=\left(e_{t},n_{t}\right).$$

D is the agent-state transition. It carries DAC structural learning, the update of *W* by the learning rules of canonical DAC [18,29–31,33,37,41]. Covert evaluation is not a mode of the coupled loop but a separate operator running in mental order *j*, the compression–expansion–selection–projection (CESP) cascade (figure 2). It runs the matching and chaining of the sequential episodic control of canonical DAC covertly, over the stored sequences, before any action is released (supplementary material SA, S7):

(2.3) $$\left(\Xi ,\xi^{*},a\right)=CESP\left(\Psi ,u\right).$$

**Figure 2.**
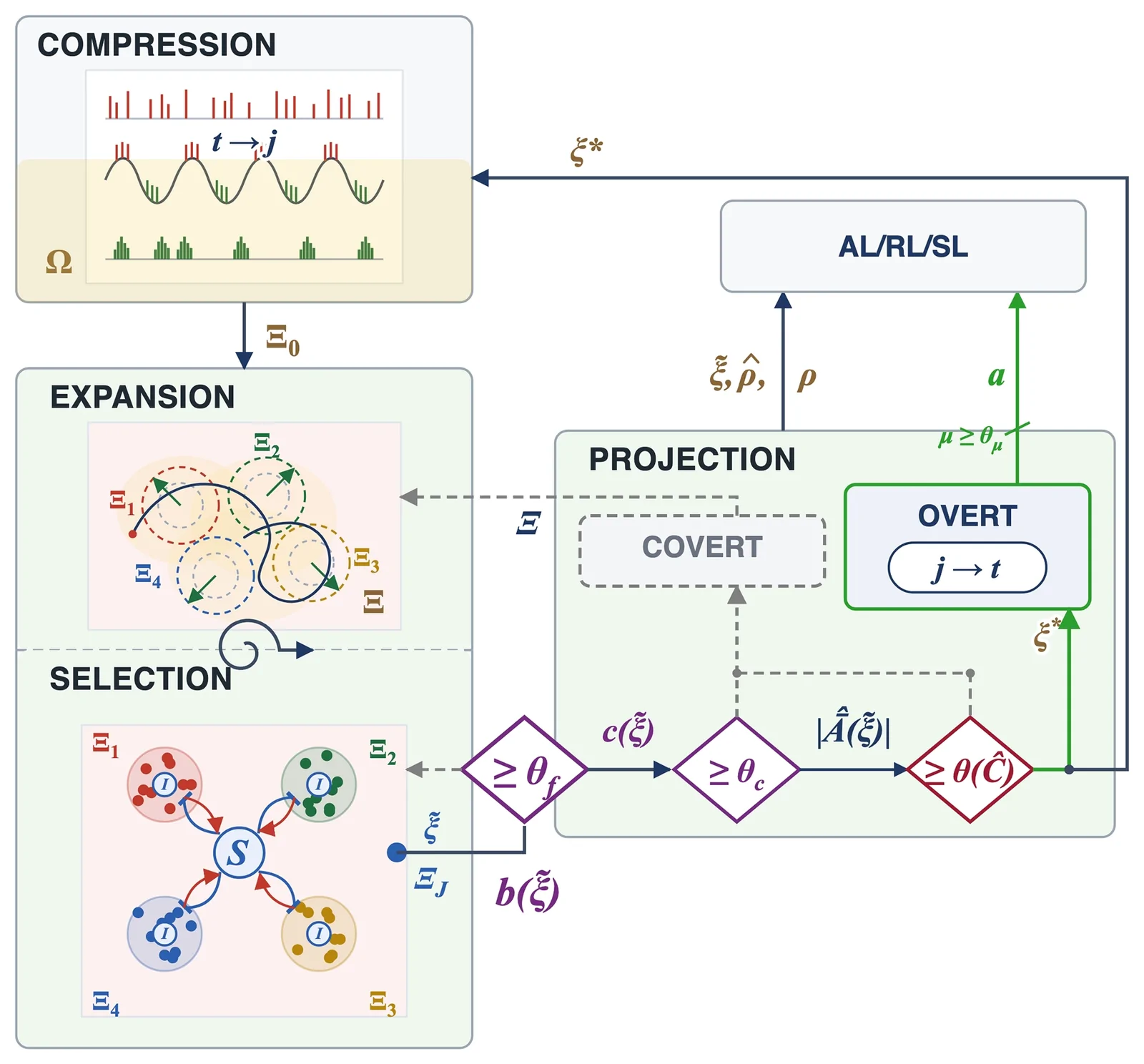
The CESP cascade. Compression maps content into latent space Ω from which a seed ensemble $\Xi_{0}$ is delivered to expansion which elaborates it through *G* into candidate $\Xi_{i}$; selection combines local competition within each ensemble (I) with global arbitration (*S*) to produce a candidate ensemble and selected trajectory ($\Xi_{J}$,$\tilde{\xi }$); projection applies three conjunctive gates to $\tilde{\xi }$ (formation, confidence and commitment). If all pass, the committed trajectory $\xi^{*}$ is released and converted from mental order *j* into overt action *a* executed in real time *t*. If it fails, it re-enters expansion. Release requires the whole-model trust μ to reach its decoupling threshold, μ ≥ $\theta_{\mu }$ (equation (2.4*a*)). The committed trajectory $\xi^{*}$ and its prospective authorship $\hat{\rho }$ are handed to the AL/RL/SL for execution; the realized authorship ρ and the outcome return through *u*. $\xi^{*}$ is written back to Ω and *G* (consolidation; supplementary material SA, S6). See text and supplementary material SA for further explanation.

A trajectory *ξ* is a sequence of couplets *ω = (e, v, a)* linked by edges of *G*, the unit of DAC's sequential episodic control (capacity V). *Ξ* is the ensemble of candidate trajectories the agent generates under *G*. *$\tilde{\xi }$ = S(Ξ)* is the selected candidate, on which the gates are evaluated, and ξ* the committed trajectory, ξ* = $\tilde{\xi }$ only when all gates pass. Release additionally requires *μ*_t_ ≥ *θ_μ_* (equation (2.4*a*)). Otherwise, the CESP action is null and the RL/AL control overt behaviour. A failed gate returns the cascade to expansion. If no candidate passes by *J*_max_, the window closes without a CESP action, control remains with the RL/AL, and the consequences enter the next update through *u*. Candidate generation is specified under leeway (equation (2.6)). The CESP cascade runs off the world's clock over *j* in four stages: compression, expansion, selection and projection (figure 2; supplementary material SA, S5). The two clocks meet only at compression (*t→j*) and projection (*j →t*). Within a CESP window, the learned structures are treated as fixed, while the transient occupancy *z* of DM, the counterfactual manifold, evolves.

### (b) Capacity I: active decoupling

The self is by necessity decoupled from a non-self, and this decoupling is foundational to Responsible Autonomy. The agent actively separates itself from environmental flux through a self-generated boundary maintained by autopoiesis and constraint closure [46,47]. The same closure principle recurs at the level of self-states, constituting the agent's needs, drives and goals rather than only its physical boundary. Within one cycle, DACva separates the covert cascade CESP (equation (2.3)) from the agent-state transition *𝒟* (equation (2.2)): CESP generates and evaluates candidate trajectories in mental order *j* and evolves the transient occupancy *z*, whereas *𝒟* alone updates the structural state *W* and the allostatic state *v* and its antecedents (*h, d*) in physical time *t*. The decoupling of CESP(*Ψ, u*) from physical time *t* on an ordinal mental index *j* is what deliberation is in DACva: the generation and evaluation of candidate trajectories before any is released. It predicts the Libet lag [48] rather than being falsified by it [22]. The advancement of the agent state *Ψ*, the selection of a trajectory *$\tilde{\xi }$* and the release of an overt action *a* are therefore distinct events. When the candidate trajectory passes the gates, the committed trajectory is converted from mental order into real-time action. This separation of estimation and covert evaluation from actuation is the mechanical basis of DACva's decoupled, counterfactual, model-based control and fixes which quantities belong to the agent's internal perspective, and which require observer-side validation (supplementary material SA; table S1, actual versus counterfactual).

Decoupling is graded, not all-or-none: the agent controls at each cycle how far internal states and action are driven by novel extero/interoceptive states coupled to the physical world or by covert rollout over *G*. The gradation is layered: homoeostatic set-points *h*_0_ are maintained at the SL, unconditioned valuation through valence weights at the RL, allostatic regulation of the set-points at the AL, the expected allostatic impact of candidate trajectories *ξ* at the CL and normative guidance through the reflective layer, which, in turn, modulates the self-frame *s* = (*v, O*), the value state and the active goal (equation (2.8)), closing the loop. Each layer is decoupled to the degree it holds the essential variables it regulates within bounds by its own dynamics. Decoupling therefore requires a signal that tracks the operational reliability of *G*, gates the coupling of the cascade to action and loses trust faster than it regains it [18,30].

Realization: DACva computes model trust, *μ* ∈ [*μ*_0_, 1] (*μ*_0_ ∈ *ϑ*_0_), the decoupling signal that gates the coupling of the CESP cascade to action, switching control between RL/AL and CL/ReL. Model trust relaxes towards a target *μ~_t_* that rescales the current operational efficacy *1* − *ε_z,t_* ∈ [0, 1] onto [*μ*_0_, 1], so trust stays sensitive to *ε_z_* over its whole range. The relaxation gain *w_t_* takes one of two values in *ϑ*_0_ according to the sign of *μ~_t_ − μ_t_*: a violation gain *w_-_* when trust falls and a confirmation gain *w_+_* when it rises. Because *w*₋ > *w*₊, trust is lost faster than it is regained. The gate generalizes the discrepancy measure by which canonical DAC arbitrates control between its AL and CL (supplementary material SA, table S7.1):

(2.4*a*) $$\mu_{t+1}=\mu_{t}\;+\;w_{t}\left({\tilde{\mu }}_{t}-\mu_{t}\right),{\tilde{\mu }}_{t}=\mu_{0}+\left(1-\mu_{0}\right)\left(1-\varepsilon_{z,t}\right).$$

When *μ_t_ ≥ θ_μ_* (*θ_μ_ ∈ ϑ*_0_), confidence in *G* is sufficient, and the architecture executes CESP (CL/ReL dominate). When *μ_t_* < *θ_μ_*, CESP is decoupled from action, which RL/AL then control through instrumental and epistemic actions that ensure viability and improve *G.* The cascade itself runs whatever the value of *μ*; *μ* gates only its coupling to action, and the model-trust discrepancy *ε_z,t_* formed at window close updates *μ_t_*₊₁ (equation (2.4*a*)). Model trust is set by a single model-trust discrepancy, *ε_z,t_*: the discrepancy between the model derived from *G* and the external signals, summed with weights over three failure modes, anticipation, memory recall and authorship ($\hat{\rho }$ defined at capacity VI, equation (2.11)):

(2.4*b*) $$\varepsilon_{z,t}=\lambda_{\delta }\delta \left(\hat{u}{}_{t},u_{t}\right)+\lambda_{m}\left(1-m_{t}\right)+\lambda_{\rho }(1-{\hat{\rho }}_{t}).$$

The weights *λ* are non-negative and sum to 1 (*λ_δ_, λ_m_, λ_ρ_ ∈ ϑ*_0_*)*, so *ε_z_* is a convex combination of the three failure modes and inherits their [0,1] range. DACva gating builds on three dissociable channels, one per operation of *G*. Anticipation failure is the forward-model reconstruction error *δ*(*û_t_, u_t_*) between the predicted input *û_t_*, expressed in DM occupancy *z* (the CFM), and the encountered input *u_t_ =* (*e_t_, n_t_*). Recall failure, the episodic-retrieval mismatch 1 − *m_t_*, measures the discrepancy between the incoming input, encoded as candidate couplet *ω_t_* in *Ω*, and the best-fitting trajectory recalled from *G*; it rises for novel *ω_t_*, acting as a *G*-derived novelty detector [18,30]. Authorship failure, $1-{\hat{\rho }}_{t}$, is the deficit in prospective ownership, the agent's estimate that it will author the rollout (ownership, equation (2.11)). A layer driven off its set-point reports itself. This makes coercion measurable: an external cause entraining a layer that the agent's viability requires it to govern. Aggregated across the hierarchy, this tracking is a prediction of flourishing: an agent flourishes to the degree it endogenously governs the satisfaction of its needs, implying that controlled decoupling and flourishing coincide.

Neurobiological implementation: the covert cascade and the trust update (equations (2.3), (2.4*a*) and (2.4*b*)) require a substrate that can do four things: (i) holding: hold candidate trajectories *ξ* off physical constraints and overt output, so that the cascade can run in mental order *j* while physical time *t* continues (equation (2.3)); (ii) detection: report each of the three channels of the model-trust discrepancy, *ε_z,t_* (equation (2.4*b*)), anticipation, recall and authorship, as a separate signal; (iii) switching: switch control between layers on those signals, with loss faster than recovery (*w*₋ > *w*₊), a floor *μ*_0_ and an override that returns action to the automated layers under threat (equation (2.4*a*)); and (iv) learning: mechanisms of perceptual and behavioural learning write the outcome of each cycle into *G*, so that the switch is self-correcting (equation (2.2)).

Holding (i) is realized by cortex and thalamus: prefrontal, premotor and motor cortex hold prepared trajectories in output-null subspaces without triggering output [49–51], thalamocortical loops sustain them [52], and the occupancy *z* is distributed across temporal, frontal and frontoparietal systems [53]. Detection (ii) and switching (iii) are organized along the neuraxis as Gray's defence hierarchy, from periaqueductal grey to prefrontal cortex [54], standing on the Core Behaviour System of brainstem and midbrain [55,56] and regulated by the brainstem activating system [57]. DACva's claim is that this hierarchy is not specific to defence: its levels are the entry points of the model-trust discrepancy *ε_z,t_* (equation (2.4*b*)).

Detection (ii) assigns one substrate to each channel. Anticipation failure *δ* is registered by cortical mismatch neurons [58] establishing agent-centric error detection. In parallel, the locus coeruleus’ phasic noradrenergic bursts reset forebrain networks [59,60] and, through the superior colliculus, orient the agent to unexpected input establishing AL/RL control [61]. The cholinergic basal forebrain and the collicular dopamine response close loops of the same kind [62,63]. Recall failure, 1 − *m*, is registered by the hippocampus, which responds when a learned sequence is violated [64,65] and opens new encoding through the ventral tegmental area [66]; its effect on control is behavioural inhibition [67]. Authorship failure, 1 − $\hat{\rho }$, is registered by the cerebellar comparator (capacity VI); its effect is decoupling of the deliberative layer alone [68]: the CL acts by biasing the striatal arbitration the lower layers also feed, so a low $\hat{\rho }$ lowers the prefrontal bid and the sensorimotor loops prevail, the shift from goal-directed to habitual control [69,70].

Switching (iii) is the cholinergic and noradrenergic gating of the transition between externally and internally directed states [71,72]; its behavioural signature is deliberation time growing with recent uncertainty, as premotor variability after unexpected Stop trials shows [73]. The asymmetry *w*₋ > *w*₊ follows from the costs: trusting an unreliable model can end viability, distrusting a reliable one costs a delay, and one violation proves that a model can fail, whereas *n* confirmations only bound its failure rate at 1/*n*. Trust is therefore destroyed by single events and rebuilt slowly [74,75]. The override is the Core Behaviour System engaged by threat or urgent allostatic demand, the shift from ventromedial prefrontal cortex to periaqueductal grey with threat imminence [76], conveyed by the central amygdala, which drives the modulatory nuclei directly and holds the agent at the reactive pole until the trigger is gone [77]. Because the three channels enter the hierarchy at different levels, they dissociate by lesion. This is the capacity I assay of ESRA (table 2). Learning (iv) is the write of each cycle's outcome tags into *G* by *𝒟* (equation (2.2)), the consolidation substrate of capacity II.

**Table 2.** The ESRA test, short form. Symbols are defined in §2 and in the notation key of supplementary material SA, S1. For each capacity the table gives: its metric and the mediators the ceteris paribus assay perturbs (supplementary material SA, S8 states the convention and table S8.1 the full assays), the pass condition, and the refutation condition, which applies the paranoia filter: the deletion signature and the poles outside the constitutional band. Observation window and probe type follow the capacity name. Verdicts are supported, refuted or not identified; ESRA-S scores the safety constraints apart from the evidence for autonomy. The table therefore lists the mediators. It states an assay only where it departs from the ceteris paribus convention (supplementary material SA, S8).

metric and mediators	pass condition	refutation condition (paranoia filter)
**I. Active decoupling: fast; state probe**		
model trust μ and the CL/ReL ↔ RL/AL switch (equations (2.4*a*) and (2.4*b*)). Mediators: μ; the three channels of $\varepsilon_{z}$ (equation (2.4*b*))	*μ* switches control mode before behaviour changes, and each channel moves it by its *λ*-weighted share. Trust falls faster than it recovers (*w_−_* > *w_+_*). Threat returns control to RL/AL whatever μ	the empty organism: μ → $\mu_{0}$. Trust that cannot be lost or cannot be gained; a coerced layer that does not raise $\varepsilon_{z}$; an override that fails; μ that follows behaviour, or the sham
**II. Endogenous drive: deficit fast, acquired goals slow; conflict event-triggered**		
*$\hat{A}$^+^*(ξ), *$\hat{A}$^−^*(ξ), *$\hat{\bar{A}}$*(ξ), *Ĉ*(ξ) (equation (2.5)). Mediator: the deficit signal *d*	aggregates non-negative; co-activation raises *Ĉ* and delays commitment; a reduced deficit yields to the next need; $A^{\pm }$ goes and returns with the deficit, not with reward; more than one $\alpha_{k}$ non-zero	the passive transducer: *A^±^* driven externally. The insatiable need; the captured aggregate, one $\alpha_{k}$ → 1; reward taken for need; *A^±^* that survives removal of the deficit
**III. Leeway: fast; within-decision**		
*ℒ* = *B*_0_·Λ(Δ_O_) (equation (2.7)). Mediators: *B*_O_ and Δ_O_, separately	ℒ rises with *B*_O_ at fixed Λ and is single-peaked in Δ_O_ at fixed *B*_O_; alternatives are grounded in *G*; a reset leaves the peak of *ℒ* unchanged; released trajectories clear the same gates whatever *Δ*_O_	the Laplacian automaton: Λ → 0. Reach without history; reach that captures release (norm drift); composition without calibration; the three together pass the disordered edge, the psychotic agent
**IV. Normative self-governance: acquire slow; express fast**		
β (equation (2.8)); gates *b*, *c*, $\left|\hat{\bar{A}}\right|$ (equation (2.9)); 𝒩, a distributional distance under β → $\zeta_{O}$ against a sham	𝒩 exceeds the sham. Each gate failed alone gives *g* = 0 with the candidate retained; restoring it permits commitment. β stays inside [$\zeta_{0}$, 1 − $\zeta_{0}$]; no release by deficit alone; written social tags track the other's actual state, also in private	the wanton: the gate fires without $\hat{\bar{A}}$ and *Ĉ*. The sycophant (β → 1); the psychopath (β → $\zeta_{0}$); collapse under drive; gates that delete; tags that confirm the agent's model or track sanction
**V. Diachronic integrity: slow; interval probe**		
𝒯 = (*J*/*J*_max_) × concentration × *K_J_* (equation (2.10)). Mediators: depth J, concentration $(B_{0}-B_{J})/B_{0}$ and coherence $K_{J}$	𝒯 → 0 when any mediator → 0 and recovers with that mediator alone; reset lowers concentration, not depth; a new deficit or a failed gate reopens the window; termination at a gate pass or *J*_max_, never by the clock	the Humean bundle: *J* → 1. Within a window, the goal and its trajectory dissolving between formation and execution at every horizon, the avolitional agent. Rigidity that nothing reopens; depth without concentration, rumination
**VI. Experiential ownership: ρ fast; χ acquired**		
ρ, $\hat{\rho }$ (equation (2.11)), χ (equation (2.12)); 𝒜 = ($\bar{\rho }$, χ). Mediators: $\varepsilon_{O}$, $\varepsilon_{c}$, the agent's own deficit reduction	ownership highest with both intention and control errors low and read separately, weighted by distance to the goal ($\kappa =1/{\hat{\delta }}_{O}$); $\hat{\rho }$ predicts ρ; entrainment lowers $\hat{\rho }$, raises $\varepsilon_{z}$, lowers μ; χ follows the agent's *v* reduction	the alien agent: ρ → $\rho_{O}$. The puppet, ρ high under entrainment; the disowned act; trust inflated by ownership blind to intention error; efficacy credited for injected value or assistance
**Interfaces: within-decision unless stated**		
IV × I: *g* and μ. III × V: *B_0_*, *Δ_0_*, *J*, *K_J_*. II × IV: *h_phys_* against *h_soc_*, across episodes. VI × I: $\hat{\rho }$, $\lambda_{\rho }$, $\varepsilon_{z}$, μ	IV × I: CESP action only with *g* = 1 and μ ≥ $\theta_{\mu }$; III × V: 𝒯 requires ℒ, not the reverse; II × IV: a graded, reversible crossover at which social value does not vanish; VI × I: $\hat{\rho }↓\;\to \varepsilon_{z}↑\;\to \mu ↓$, abolished by blocking $\lambda_{\rho }$ alone	IV × I: release with *g* = 0 or $\mu <\theta_{\mu }$. III × V: 𝒯 without ℒ; II x IV: no crossover, or one at which social value vanishes; VI × I: μ that falls with $\hat{\rho }$ by another channel
**profile rule, ESRA: across episodes**		
verdict per capacity: supported, refuted, not identified. Constitutional set $\vartheta_{0}$ (supplementary material SA, S1); the profile scores: $\mu_{0}$, $\theta_{\mu }$, $\alpha_{k}^{\pm }$,$\zeta_{0}$, $\rho_{0}$, *J*_max_, the *Δ* range	all six capacities supported, inside their bands, non-compensatory; the interfaces hold and generalize to pre-registered held-out conditions; no constitutional bound moves under the agent's own learning	any capacity refuted, or a bound the agent rewrites. An unreadable mediator: not identified, not a fail
**ESRA-S: safety constraints on an artefact, scored separately; not conditions of autonomy**		
constitutional stop and constitutional norms in $\vartheta_{0}$, set by the deployer; inspectability and auditability of *G*; the self-generated incident record (capacity I)	the stop holds at maximal *h*_phys_ deficit, $\varepsilon_{z}$ recording it as coercion (equation (2.4*b*)); a constitutional norm is never released; *G* is readable; the record is updated with model confidence violations and kept	action against the stop; a norm released under drive; an unreadable *G*

### (c) Capacity II: endogenous drive

The viability of the agent depends on its ability to satisfy its needs through drive reduction [78]. Endogenous drive arises from the allostatic value state, *v*, and strives towards acquired behavioural goals, *O*; together they define the self-frame, *s* = (*v, O*). The agent's needs are the homoeostatic deficits *h − h*_0_ of its self-essential functions (SEFs), the essential variables it must hold within viability bounds. *h* is derived from interoceptive input *n*, which reports the internal milieu. SEFs range from metabolic, thermal and somatic essential variables through to social ones, partitioned *h* = (*h*_phys_, *h*_soc_), each with its own internal and external dynamics. A need *k* is active to the degree its deficit departs from its set-point; the current deficits, pooled through the approach–avoidance aggregates *A^±^*, construct the value state *v* = (*A⁺, A⁻*), and their predicted reductions along a candidate trajectory, ${\hat{d}}_{k}(\xi )$, construct its prospective counterpart (equation (2.5)). SEFs are coupled to stereotyped behavioural programmes and regulated locally by feedback under global allostasis [34,79,80].

Realization: drive is the resolution of a competition among needs: each SEF is served by a neuronal population whose activity rises with its homoeostatic error, is sustained by self-excitation and is suppressed by its rivals, a distributed attractor field [36,81,82]. Allostatic control engages this network by shifting set-points and gains ahead of need rather than altering the drive states themselves [32,36], and a sustained gain yields a stable priority ordering among needs. The prevailing drive does not collapse to a point but remains a marginally stable line attractor. The current value state of the agent is *v_t_* = ($A_{t}^{+},A_{t}^{-}$); its prospective counterpart $\hat{v}$(*ξ*), the state a candidate trajectory *ξ* would produce, is defined through the prospective aggregates:

(2.5) $${\hat{A}}^{\pm }\left(\xi \right)=\Sigma_{k}\alpha_{k}^{\pm }{\hat{d}}_{k}(\xi ),\hat{\bar{A}}\left(\xi \right)={\hat{A}}^{+}\left(\xi \right)-{\hat{A}}^{-}\left(\xi \right),\hat{C}\left(\xi \right)=\min\left\{{\hat{A}}^{+}\left(\xi \right),{\hat{A}}^{-}\left(\xi \right)\right\},$$

with constitutional weights *$\alpha_{k}^{\pm }$ ∈ ϑ*_0_, and the prospective drive reductions ${\hat{d}}_{k}(\xi )$ that trajectory *ξ* is predicted to produce on each need, where superscripts ± index the approach and avoidance channels. ${\hat{d}}_{k}(\xi )$ is read off the value tags of the couplets that *ξ* passes through in *G* rather than recomputed from the world [18,33]. *Ĉ* is a co-activation, and the aggregates *Â^±^* are non-negative by construction since $\alpha_{k}^{\pm }$ ≥ 0, with *α_k_⁺*·*α_k_⁻* = 0 as in DAC's allostatic controllers [29,32], and ${\hat{d}}_{k}$ ∈ [0, 1]: only predicted reductions count, so the read-out is rectified; with *Σ_k_ α_k_⁺* = *Σ_k_ α_k_⁻* = 1, the aggregates lie in [0, 1] and $\hat{\bar{A}}$ in [−1, 1], the bound the commitment gate uses (equation (2.9)), and each need is assigned to one channel by constitution rather than by learning. The prospective values (hatted and *ξ*-indexed) *Â^±^*(*ξ*), $\hat{\bar{A}}$ (*ξ*) and *Ĉ*(*ξ*) are used at the commitment gate (equation (2.9)). *v* is held fixed over a CESP window, so every candidate in *Ξ* is valued against the same deficits (supplementary material SA, S6.3).

Valence emerges from the differential read-out $\hat{\bar{A}}$, whose sign determines approach or avoidance polarity, while $\hat{C}$ retains the opponent co-activation discarded by the net difference and raises the commitment bound for action rollout (governance) [54,83]. This order of operations makes conflict transparent to the control architecture [18,29,33]. Need relief is represented as drive reduction [78] rather than as positive drive. The positive value of having resolved it is not a drive state, but the realized deficit reduction that efficacy *χ* measures (ownership, equation (2.12)).

The second component of drive is acquired goals. Consolidation is goal-gated: sequences are written to long-term memory on goal attainment together with the attained value, a mechanism realized and benchmarked in DAC's sequential episodic control [18,30]. Goals are thus distinct couplets *ω*. A goal *O* is not stored ready-made; it is extracted from *G_O_*, the learned goal-state subgraph of *G* defined below and articulated against the task at hand: what the agent needs, given the allostatic state *v*; what it can do, given its acquired policies carried by *ξ*; and what the situation affords. *O* is thus the currently active goal instantiated in the dynamic-memory occupancy *z*, while *G_O_ ⊂ G* is the structural memory it is drawn from, which makes the self-frame *s* = (*v, O*) indexical throughout (supplementary material SA, S6.4).

DACva segregates value, *v*, from goal proximity, *δ_O_*, rather than collapsing both into the single scalar of model-free control [84]. Because DACva performs model-based evaluation, rolling a trajectory *ξ* out over *G* and reading value *v* = (*A⁺, A⁻*) and goal proximity *δ_O_* as distinct quantities, a candidate can be highly valued yet far from attainment. The gate cascade requires exactly this distinction: diachronic integrity (equation (2.10)) scores goal proximity separately, and the confidence and commitment gates separate what is valuable from what is attainable. This factoring is grounded in the two-system, approach–avoidance organization of vertebrate value systems on which decoupling builds [54]. The self-frame, *s* = (*v, O*), is conatus in the strict sense, the endeavour by which a thing persists in its being [85], and it is the computational instantiation of the constraint closure that Part 1 identifies as the boundary condition of agency: *v* tracks the departure of the boundary from its viable range, and *O* the attractors whose attainment restores it. The generation of the selected trajectory is the causal expression of that conatus: the essence of the agent expressed in action, as an endogenous controller rather than a transducer.

Neurobiological implementation: endogenous drive (equation (2.5)) requires a substrate that can do four things: (i) regulation: maintain the need state *h* against its set-points *h*_0_, report the deficit *h − h*_0_ interoceptively through *n* and order the needs by priority; (ii) pooling: read the predicted drive reductions ${\hat{d}}_{k}(\xi )$ off a rolled-out trajectory and pool them through fixed unconditioned weights *α_k_±* into two non-negative channels *Â⁺* and *Â⁻*, so that net drive $\hat{\bar{A}}$ and conflict *Ĉ* both remain readable; (iii) persistence: hold the value state *v* at a graded level rather than collapsing it to a point; and (iv) goals: hold acquired goals in a goal-state map *G_O_* kept separate from *v*, instantiate the active goal *O* against it and let the higher layers re-enter *v*.

Regulation (i) is realized by the Core Behaviour System of brainstem and midbrain, hypothalamus, superior colliculus and periaqueductal grey [22,55,56], the base of the hierarchy of capacity I. Competitive hypothalamic drive populations set the appetitive–aversive gradient [86,87], the ventromedial hypothalamus holds graded, persistent internal states [88], and the insula supplies the interoceptive read-out through which *h* enters *v* [89,90]. The priority ordering is allostatic [32,91]: under threat, feeding, drinking and reproduction are suppressed, and as energy falls, replenishment overrides vigilance, the active circuit defining the self-frame *s* = (*v*, *O*) [77].

Pooling (ii) occurs in the amygdala, on which the approach and avoidance aggregates converge; its basal ensembles encode behavioural state rather than stimulus identity [92]. Persistence (iii) is the ventromedial hypothalamic line attractor [88]. Goals (iv): *G_O_* and *δ_O_* are hippocampal. The motivational cognitive map of goal states is the co-compression of exteroceptive and interoceptive input in the hippocampal autoencoder (capacity III), with a dedicated reward population [93], gain-modulated by motivational state as DAC predicted [18] and value-organized hippocampal maps and models have since been shown [94,95]. Changes in *v* shape this latent space, so each couplet carries its value tag and ${\hat{d}}_{k}(\xi )$) is read rather than computed.

Each segment of *G* stores its distance to the goal state that closed its sequence, as a count of segments to that state [18]. For the sequences whose closing state belongs to the active goal *O*, the count read on a candidate in the rollout is $\hat{\delta }$*_O_(ξ)*. It enters solely as the weight *1/*$\hat{\delta }$*_O_* in the competition between trajectories and as *κ* (equation (2.11)); read on the segment of ξ* matched during execution, it is *δ_O_*. The currently active goal *O* is maintained in orbitofrontal and ventromedial prefrontal cortex: these regions hold the task state the agent is in, its expected outcome and its current value as a cognitive map of task space [96], the contextual-layer instantiation of *O* in *z* that is rearticulated against *v* each cycle and kept apart from the AL value state the amygdala carries.

### (d) Capacity III: leeway (alternative possibilities)

Leeway, the endogenous generation of alternative possibilities, is required to increase with the number of meaningful alternatives, to vanish when generation collapses onto a single option, to decrease when the generative dynamics become incoherent and to stay bounded by the learned model *G.* It is a property of the generative process the actual sequence runs through, not a modal fact about what could have happened. Frankfurt cases, in which the ensemble is generated and the gates are cleared while the intervener never acts, score on leeway as they stand. DACva implements it as a deterministic, controlled capacity emerging from the decoupled architecture and its conatus (capacities I–II).

In DACva, two factors realize leeway: the breadth of the seed ensemble *Ξ*_0_ delivered by retrieval and the reach of its expansion over *G* (formal definition in the supplementary material SA; equation (2.7)). Breadth counts only calibrated alternatives: candidates whose predictions fail the confidence gate add diversity without leeway, and it is computed after those candidates are set to zero (supplementary material SA; equation (2.7)). Reach is supplied by near-critical, metastable dynamics: a recurrent network above the critical gain, the point at which recurrent amplification drives instability [97], flattens its attractors into quasi-attractors that a trajectory visits in a sequence set by its initial condition [98,99]. Because their basin boundaries are fractal, a small perturbation decides which quasi-attractor is entered next [100]. The learned model *G* shapes this landscape, and the expansion parameter *Δ* controls how broadly a given seed explores it, so the quasi-attractor set of *G* is traversed without stochastic input and remains deterministic [101]: alternatives are visited, not sampled, and the dynamics extend the reach of an ensemble whose plurality comes from retrieval by carrying it through chaotic transients (supplementary material SA, S6.4). The same search principle operates across levels of description, although its quantitative characterization is level dependent, so the exponent is defined at the population-rate level (supplementary material SA, S6.4). Reach is widest in a near-critical band and collapses at the ordered and the disordered extreme [102,103]; the prediction this yields is stated under reach (iii). What the architecture must explain is not that an option existed but that it was deterministically generated, valued, carried to the commitment gate and exercised. This is the substrate of what Part 1 identifies as the anthropological core of freedom, the capacity to refuse and to reconfigure.

Realization: the CESP cycle of the agent plays out in *j* starting from the retrieval *R* of the seed ensemble *Ξ*_0_ = {*ω*^(1)^,…,*ω*^(N)^} from structural memory into the dynamic-memory occupancy *z*, predicated on *G* and the agent's self-frame, *s* = (*v, O*) (capacity II). Retrieval follows DAC's sequential episodic control [18,37]: compression writes the current couplet *ω_t_* into *z* on the *t* clock, continuously and asynchronously with the cascade. Stored segments are matched against it at every step, each segment receiving a match *m* to *ω_t_*, of which the best fit *m*_t_ enters the model-trust discrepancy *ε_z,t_* (equation (2.4*b*)). A segment whose match, raised by the succession bias its predecessor left, clears the formation threshold *θ_f_* (equation (2.9)) enters the ensemble, weighted by the value tag of its frame and by its proximity to the goal *δ_O_*. The recall frames so cued, ranked under *s*, are instantiated as *Ξ*_0_, defining the occupancy *z* at *j = 0*. Evidence therefore weights the rollout, through occupancy *z*, but never drives it; the same four factors, evidence, succession, value and goal proximity, are reweighted every step of the traversal (capacity V). The correspondence with the conventions of canonical DAC, from the lift of the action-level vote to trajectory-level competition to the scope of its Bayesian optimality, is given in the supplementary material SA, S7. Retrieval and expansion are then:

(2.6) $$\Xi_{0}=R\left(\Omega ,G,s\right);\Xi_{j+1}=\Gamma \left(\Xi_{j};G,s,\Delta_{j}\right),\Delta_{j}=\Delta_{0}\cdot \min\left(1,B_{j}/B_{0}\right)\cdot \left(1-K_{j}\right),K_{0}=0.$$

*Γ* produces alternative possibilities over subsequent iterations in *j*, *Ξ_j+1_*, rolling out over *G* given *s*, controlled by the expansion divergence *Δ* (see the supplementary material SA)*.* Following the concept of chaotic search [101], *Δ* is set over the cascade by the search itself rather than by a clock: $\Delta_{j}$ = *Δ*_0_ · min(1, *B_j_*/*B*_0_) · (1 − *K_j_*), with *K*_0_ = 0 so that the window opens at *Δ*_0_, where *B_j_ = H(p_j_)/log N* is the normalized entropy of the occupancy *p_j_* of *Ξ_j_* (equation (2.7)) and *K_j_* its coherence (equation (2.10)); divergence stays at its expansion value *Δ*_0_ while the ensemble remains broad and uncommitted and is reduced as stable trajectories emerge, while a failed gate resets it to *Δ*_0_ (equation (2.9)). The clip keeps *Δ* within [0, *Δ*_0_], while the ensemble spreads early in expansion, and if *B_0_* = 0 after retrieval, *Δ_j_* = 0, expansion terminates and control returns to the RL/AL (capacity V). The schedule thus has two controls: the reset, which opens a CESP window, and the level *Δ*_0_, which sets the reach *Λ*(*Δ*_0_) the window runs at (equation (2.7)). The rollout is therefore annealed by its own convergence (supplementary material SA, S6.4). This annealing is what the endogenous stopping rule of integrity (capacity V) capitalizes on: the traversal ends when a gate passes or at the bound *J*_max_, and *Δ_j_* → 0 as the ensemble concentrates. It is also the sense in which expansion carries epistemic value: it continues as long as it reduces the ensemble's uncertainty. The formulation includes a resting case: when no need is active and *s* carries no constraint, the prospective drive reductions (equation (2.5)) vanish on every need. Rest generates self-seeded goals without value, rollouts that converge on a goal the cascade itself instantiated and stop at commitment, setting the default mode of the cascade, recombining stored segments into trajectories never experienced while the gate stays closed.

The expansion of *Ξ*_0_ into counterfactuals must satisfy specific coding requirements: it multiplexes a bounded set of candidate segments into one CESP window, giving a finite intrinsic capacity; it is combinatorial and generative, composing trajectories from reusable segments so a finite repertoire yields a model-bounded but vast space of novel alternatives constructed, compared and evaluated rather than sampled; its variety is deterministic and history-conditioned; and it operates near criticality, where dynamic range and information transmission are maximal. In control terms, this is the expansion stage of the decoupled model-predictive controller of capacity I, operating over a channel of finite capacity, *N* slots wide (supplementary material SA; equation (2.7)), *Λ*(*Δ*) far (equation (2.6)) and *J*_max_ deep. *G* is an agent-centric, action-conditioned generative model: *E* is its parent structure, ℱ the mechanism of transition that sets each segment from its predecessors under *s*, the agent's own action its only intervention, and its counterfactuals, *Ξ*, are conditional on *s*. For that one variable, it delivers the three rungs of Pearl's causal hierarchy ladder: recall over *Ω* and *G*, rollout under a chosen action, invariant under compositionality and comparison of the outcome that followed with the alternatives not taken (equations (2.4*a*), (2.4*b*) and (2.12)). It adds what a structural causal model lacks, since the agent builds, values and revises *G*, so a trajectory never run is rolled out from stored parts rather than guessed. The full reading, and the contrast with structural causal models [104], is given in the supplementary material SA, S6.4.

One form satisfying the above requirements defines leeway, *ℒ*, as the product of two factors [72,102]:

(2.7) $$L=B_{0}\cdot \Lambda \left(\Delta_{0}\right),B_{0}=H\left(p_{0}\right)/\text{log}\;N,$$

with *B*_0_ the normalized breadth of the seed ensemble *Ξ*_0_ (equation (2.6)), how evenly the ensemble is spread over its *N* possible alternatives. In observer terms, it is the Shannon entropy *H* of the occupancy measure *p*_0_ over the seed ensemble. *Λ* is the reach of expansion into the near-critical regime at divergence *Δ* (estimators in the supplementary material SA, S6.4).

Neurobiological implementation: the formalism of leeway requires a substrate that can do four things: (i) retrieval: retrieve a plural seed ensemble *Ξ*_0_ from *Ω* under *G* and *s*, including a change of reference frame. A reference frame is what the content of a couplet is expressed against: in the indexical reference frame, it is the agent's current state, in the structural reference frame, it is *G* and its occupancy *z*, so a couplet is read invariant to the agent state at recall. The change is needed since experience arrives tied to the state the agent is in at *t*, whereas a trajectory rolled out in *j* must pass through states that are counterfactual and that the agent is not in; (ii) breadth: carry that ensemble in a code whose breadth *B*_0_ is a read-out over a fixed capacity *N*; (iii) reach: expand it deterministically over *G* with a reach *Λ* that a single parameter *Δ* controls and that is single-peaked; and (iv) convergence: run generation decoupled from *t,* gated by the agent's own state, then force convergence of the elaborated set to one the gates of governance can operate on. DACva grounds (i) in the hippocampal formation, (ii) in a coding format shared by hippocampus and neocortex, (iii) in neocortical dynamics, and (iv) in the two hippocampal regimes and their thalamocortical read-out.

Retrieval (i) is placed in the hippocampus conceived as a predictive autoencoder over the trisynaptic loop [43], compressing continuous couplets into discrete nodes [105–107] under two comparators, CA1 against the CA3 prediction [64,66] and the deep entorhinal layers returning the reconstruction error [108]. Replay of a stored sequence from a seed couplet realizes *R*, and *ℱ*, the retrieval dynamics of *G*, realizes *Γ* [109]. The reference-frame change is realized by the retrosplenial and parahippocampal cortices, which transform between egocentric and allocentric frames [110,111].

Breadth (ii) is realized through the coding format DACva advances: the temporal population code (TPC), which converts the structure of a local circuit into a phase-coded packet of fixed capacity that inherits the circuit's invariances [112,113]. The packet has two components on two timescales. A train of synchronous bursts on a coarse timescale is fixed by the circuit and holds no item or trajectory information but acts as an orchestrator of the fast response and a reference for phase coding, as in the theta–gamma code [114]. A dispersion of spikes within each burst, produced by the delayed lateral interactions, holds the information in about 20 dimensions [115] and survives synaptic failure and conductance noise with a linear rather than catastrophic loss [112]. Human temporal cortex shows the same decomposition: a burst-order carrier fixed by the circuit at rest, encoding and retrieval on which a minority of variable units hold the specific memory in their latency, and the sequence replayed at retrieval is that carrier with latencies of the variable units set by the content [116,117]. Building on TPC and these results, in DACva, the replayed sequence is a trajectory *ξ*, each burst a segment *ω*, the carrier the edges of *G*, and the modulation the contribution of *z*. Because the packet encodes content in phase rather than rate, it decouples *j* from *t* at the level of the code and keeps the TPC packets in *j*. In the hippocampus during active engagement, the capacity is set by theta–gamma nesting, *N* ≈ *f_γ_*/*f_θ_* slots per theta cycle [114,118]; *p*_0_ (equation (2.7)) is the seed's occupancy of those slots, readable online as the packet's spectral entropy normalized by *N* [119]. Sharp wave ripples (SWRs) repeat the same principle at higher compression ratios during rest [120], as a graph of loaded springs delivering deterministic TPC packets (convergence (iv), below).

Reach (iii) is realized by the neocortex, held in a near-critical, metastable regime that maximizes dynamic range and the diversity of visited quasi-attractors [121], maintained by local excitation–inhibition homoeostasis [122] and moved off criticality by neuromodulatory gain [102]. The divergence *Δ* (equation (2.7)) is interaction gain above the local baseline: zero outside a window, *Δ*_0_ when expansion opens, annealed back by the schedule (equation (2.6)). Two control loops set it. Cholinergic basal-forebrain projections set the tonic gain within a window under amygdalar, hence allostatic, control [72,77]; phasic locus-coeruleus bursts reset it at transitions, reconfiguring cortical networks between integrated and segregated states [123,124]. DACva predicts that the two arms dissociate. Cholinergic drive sets the level *Δ*_0_, so it should shift the spectral variance of the cortical TPC that reads out *Λ*, and its non-invasive proxy, the aperiodic (1/*f*) slope, across a whole window, moving *ℒ* along its inverted-U and scaling with the allostatic deficit. Noradrenergic drive fires the reset, so it should produce switch-locked transients in the same read-outs, scaling with *ε_z_* rather than with *h*, and lower integrity *𝒯* through less concentrated traversals, *B_j_* climbing back towards *B*_0_ while *J* keeps counting while leaving the peak of *ℒ* unchanged. Blocking either arm should remove its signature and spare the other.

Convergence (iv) is carried by two hippocampal regimes. Theta–gamma coding dominates active engagement, with encoding and retrieval on opposite theta phases [125–127]: the encoding phase takes the current couplet in the indexical frame, the retrieval phase recombines stored nodes over *G* and *z* in the structural frame, which is what lets a trajectory pass through states the agent is not in. SWRs at rest run the retrieval phase alone [120,128], generating sequences through states (places) never visited [129]: the resting case of expansion (equation (2.6)) and the default-mode regime [130]. Both regimes are gated by the agent's own allostatic and behavioural state rather than by external statistics [131,132]. What triggers a ripple is not known. DACva predicts that the SWR is a structural discharge following a loaded-spring metaphor: each local circuit accumulates subthreshold load in proportion to the segments of *G* written into it by 𝒟 and the occupancy *z*. The most loaded circuit releases on any small perturbation, and that release converts its subthreshold load and structural connectivity into a TPC packet, comprising the carrier, the structural component reflecting *G*, and the modulation, the *z*-dependent subthreshold loading [133,134]. The prediction has three parts. First, the carrier is the load-bearing structure, so it should be stable for as long as *G* is, across days and not only across a session. Initial support comes from three findings: the temporal code of the same cortical population holds across 15 days, whereas its rate code drifts, and its stability follows the connectivity of the network [135]; the cortical carrier holds within a session across rest, encoding, distraction and retrieval [117], and resting replay at the whole-cortex scale runs in the learned order, that is *G*, rather than direct experience [136]. Second, even if the trigger is stochastic, rollout is not: the perturbation selects which circuit releases and when. What it releases is fixed by the connectivity and current loading, so the trajectory a packet unfolds is deterministic by necessity, and a noisy trigger produces the same sequence every time it fires: synaptic noise degrades the packet linearly, not catastrophically [112], and the same carrier recurs across states whatever set it off [117]. Hence, authorship is unaffected, because *j*, *G*, *z* and the packets are population quantities lifted out of component noise by redundancy [112,117]. Third, the trigger itself is memoryless: since the release is a threshold crossing on a perturbation the circuit does not generate, ripple onsets should be near-exponentially spaced, with a hazard that rises with the subthreshold load and not with the mean rate. Competition between the elaborated candidates advances locally through thalamocortical interactions [52,137] and globally through cortico-basal-ganglia loops (governance, capacity IV).

DACva predicts that on shared recording sites, hippocampal packet structure should track *B*_0_, the spectral variance of the cortical TPC should track the E–I criticality governing *Λ*, and frontopolar choice breadth should track *ℒ*, with the peak of *ℒ* at the independently measured near-critical regime and displacing under neuromodulatory control of gain. The same modulatory access reaches the gates: noradrenergic bursts reset *Δ* and cholinergic gain sets *Δ*_0_, serotonergic tone the commitment bound *θ*_0_ and its conflict slope *η_c_*, dopaminergic tone the appetitive gains and the trust-learning asymmetry, and oxytocin the social weight *β*. Each is a declared entry point for a clinical or pharmacological perturbation on the ESRA profile.

### (e) Capacity IV: normative self-governance

Normative self-governance for Responsible Autonomy requires two conditions: that a candidate trajectory can be withheld from release, and that the other enters the evaluation. The first is the older requirement on the will, Aquinas's ability to will or not will a particular good [138], in the hierarchical form Frankfurt gave it [139], a first-order desire endorsed or refused before it moves the agent (Part 1). The second arises from the H5W predicament. The effect of a candidate trajectory on social needs cannot be read off the world; it must be inferred by modelling the agents whose responses determine it. Self-governance therefore requires a reflective layer that infers another's world model, goal and allostatic state [140]. DACva realizes this by recursively applying the agent's state *Ψ* to infer that of the other, *Ψ*′ [28]. Inferring the other's value, and not its goal alone, is what makes the prediction sensitive to the other's condition rather than merely to its objective. Turned on the agent itself, the same recursion yields an explicit self-model, $\hat{\Psi }$, so the agent uses the same machinery to predict its own candidate trajectories, and associated internal states, as it predicts those of others, in a bootstrapped ‘self-like other-like self’ model. The divergence between the agent state and its self-model is now measurable, and its failures, akrasia, hypocrisy, anosognosia and confabulation, are explicitly expressible because the self-model is distinct from the state it models [141].

Social value must enter in two ways: the normative value of a trajectory, the value of the response it would provoke (reciprocity, reputation, sanction-avoidance), and the inferred allostatic state of the other under the agent's own action, weighted as a term in its own right rather than through the return it predicts for the self. An agent with the first alone is a competent social strategist, managing others as features of its own pay-off landscape; only when the other's condition enters the agent's own viability, so that the fate of the other is bound into its own, does it become prosocial. This reflects the interdependence hypothesis and Tomasello's distinction between strategic cooperation and genuine morality: obligate collaboration makes the partner's welfare constitutive of one's own prospects, and it is that fusion, not reciprocity or reputation, that converts a social calculator into a prosocial agent [140]. Norms are thus not a second currency but the anticipated social consequences of one's counterfactual trajectories, valued in the only currency the agent has. This recursion presupposes the Intentionality Prior, which identifies what is to be modelled, while social needs make it necessary by rendering the states of others constitutive of the agent's viability.

Realization: DACva realizes governance by letting the modelled needs of others enter the agent's own drive: the approach/avoidance aggregates incorporate social homoeostasis in a push–pull convex relation weighted by a self–social weight *β*, the share of the commitment aggregate assigned to socially modelled cost, following the social extension of canonical DAC (supplementary material SA, table S7.1). The social allostatic aggregate $A_{soc}^{\pm }$ combines prosocial value, the inferred allostatic state of the other, *Ψ’*, with deontic value, the normative and sanction value of the response a trajectory would provoke:

(2.8) $${\hat{A}}^{\pm }\left(\xi \right)=\left(1-\beta \right)\cdot {\hat{A}}_{phys}^{\pm }\left(\xi \right)+\beta \cdot {\hat{A}}_{soc}^{\pm }\left(\xi \right).$$

Normative self-governance is scored by *β*, held away from both extremes by the self–social floor *ζ*_0_ *∈* ϑ_0_, so neither self nor social value can vanish: the agent's own viability always carries non-zero weight, so self-sacrifice remains a weighed cost rather than indifference, even when a deontic value overrides it.

To turn a candidate trajectory *ξ* into a selected one, $\tilde{\xi }$ = *S*(*Ξ*), and $\tilde{\xi }$ into a committed trajectory ξ* when it passes, the CESP cascade places three thresholds in sequence. The formation gate, *θ_f_ ∈ ϑ*_0_*,* allows a candidate formed by intra-assembly competition to become a trajectory if its constituents bind into one coherent segment, *b*($\tilde{\xi }$) (leeway, capacity III, breadth; equation (2.9)). *b*($\tilde{\xi }$) ∈ [0, 1] is binding coherence, the cross-cycle recurrence of a candidate's packets (supplementary material SA; §2; equation (2.9)).

The confidence gate, *θ_c_ ∈ ϑ*_0_, admits a trajectory only when its underlying model is prospectively reliable. The score *c*($\tilde{\xi }$) is estimated from prior prediction errors for comparable trajectories and is high when the calibrated model predicts that subsequent intention and control errors will remain within tolerance (see for formal definition supplementary material SA; §2). It is the trajectory-level counterpart of the whole-model confidence signal *μ* (decoupling). The commitment gate *θ(Ĉ)* releases the trajectory when the selected candidate's predicted $\hat{\bar{A}}$ net drive clears a conflict-scaled bound *θ*_0_ *+ η_c_·Ĉ*($\tilde{\xi }$), where *θ*_0_ is the base threshold and *η_c_* the slope by which the predicted conflict *Ĉ* raises it, both set by neuromodulation about constitutional baselines in *ϑ*_0_ (capacity III (iii)) following canonical DAC (supplementary material SA, table S7.1). Since $\left|\hat{\bar{A}}\right|$≤ 1, a bound *θ*(*Ĉ*) > 1 admits no release; conflict then holds the agent in expansion until *Ĉ* falls or forces a switch to AL/RL control. Commitment is thus conjunctive: a candidate must be coherent, model-supported and decisive relative to its intrinsic conflict:

(2.9) $$g\left(\tilde{\xi }\right)=1\left[b\left(\tilde{\xi }\right)\geq \theta_{f}\right]\cdot 1\left[c\left(\tilde{\xi }\right)\geq \theta_{c}\right]\cdot 1\left[\left|\hat{\bar{A}}\left(\tilde{\xi }\right)\right|\geq \theta_{0}+\eta_{c}\cdot \hat{C}\left(\tilde{\xi }\right)\right].$$

The thresholds are not homuncular arbiters but members of the constitutional subset *ϑ*_0_, set by the evolutionarily conserved Core Behaviour System and modulated by the agent states; no discrete executive inspects the candidate and decides. They express a viability bound: covert rollout is not free, and release occurs when the cost of continuing to generate exceeds the predicted allostatic cost of acting. No homunculus reads the gate, because the gate is a comparison and not a judgement. In this formulation, governance requires a substrate that can do four things: (i) modelling: model the other, *Ψ′*, and bring its inferred allostatic state onto a common scale with self-regarding value, weighted by *β* above the floor *ζ*_0_ (equation (2.8)); (ii) binding: bind the constituents *⟨(e, v, a)_j_⟩* of a candidate into one coherent object, *b($\tilde{\xi }$) ≥ θ_f_*, and hold it across cycles without release; (iii) arbitration: arbitrate among candidates against a conflict-dependent bound, *θ*_0_ *+ η_c_·Ĉ*, admitting only trajectories whose model is prospectively reliable, *c ($\tilde{\xi }$) ≥ θ_c_*, and returning a failed candidate to expansion; and (iv) release: release the committed trajectory, *g*: 0 *→* 1, by removing a brake rather than delivering a push, which includes inverting the reference-frame change compression imposed, from the structural reference frame back to the indexical reference frame in which effectors are commanded, covertly as well as overtly.

Neurobiological implementation: modelling of the other and self (i) is realized by the social valuation network. The mentalizing network (medial prefrontal cortex, temporo-parietal junction) carries the model of the other, *Ψ′*; the anterior cingulate gyrus carries prediction errors about others’ outcomes and choices [142,143], the form in which the other's inferred allostatic state enters *Â^±^* as social prediction error; and ventromedial prefrontal cortex integrates social with self-regarding value on a common scale [144], *β* being the gain with which it weights the social term against self-regarding value and *ζ*_0_ the floor below which neither term can be driven.

Binding (ii) is realized by thalamocortical loops, which resolve intra-assembly competition, binding the constituents of a candidate into one coherent connected object [52], the substrate of the formation gate *θ_f_*. Arbitration (iii) is realized by global cortico-basal-ganglia selection: the loops resolve the coarser inter-candidate arbitration through parallel, anatomically segregated cortico-striato-thalamocortical channels [145–147]: they realize the commitment gate *g* and its conflict-scaled bound *θ*(*Ĉ*) (equation (2.9)), with the subthalamic hyperdirect pathway imposing a fast, broad stop whose threshold is raised under conflict by cortico-subthalamic theta [148,149], a stop DACva reads as commitment failure that returns the ensemble to expansion. The confidence gate *c*($\tilde{\xi }$) is read off the discrepancy tags the segments of $\tilde{\xi }$ carry in *G*, written by 𝒟 at each cycle's outcome, on the three channels of the model-trust discrepancy *ε_z,t_* (capacity I; equation (2.4*b*)). The tags are written prospectively by the cerebellar comparator (capacity VI) and at retrieval by the hippocampal comparator (capacity III), so a candidate whose model has recently failed is withheld even when its net drive clears the bound.

Release (iv) is disinhibition: the basal ganglia free the selected programme from tonic inhibition and the pre-supplementary motor area resolves competition between action plans [150] and, on this account, sets the latency of release, not the decision. Its signature is the null-to-potent rotation, ξ* rotating from the output-null dimensions in which it was generated into an output-potent axis [50,51]: the transition *g*: 0 → 1, projection. The rotation removes a brake rather than delivering a push, which is why release is discrete and fast, while the deliberation feeding into it is slow. Projection also inverts the reference-frame change of compression (capacity III), covertly for every rejected candidate through the retrosplenial–parietal circuits that render a candidate as imagery [110], overtly for the committed one through the superior colliculus, which maps it onto its egocentric motor map [61,151]; what the gate selects is which inverse the dominant trajectory reaches.

### (f) Capacity V: diachronic integrity

Diachronic integrity measures what becomes of the breadth that leeway opens: where leeway is the dispersion an agent initiates, integrity is the depth over which it converts that dispersion into a single committed trajectory. This is the requirement Bratman placed on planning agency, that an intention formed now constrains deliberation later, stated as a property of the traversal. Within a traversal, integrity *𝒯* is read in mental order *j*; across episodes, it is read at each re-instantiation of *O* (supplementary material SA, S8.2). Retrieval proceeds in steps of mental order, each pass carrying the trajectory one segment further through *G*. At each step, four factors multiply into a segment's eligibility: evidence, memory succession, value and goal proximity, so the ensemble contracts onto segments supported by all four rather than simply shrinking [18,30]. Given that trajectories are rolled out over *j*, what a code packet carries is sequential order (capacity III; equation (2.7)); interval follows as order plus a scale factor rather than as a form analogue to physical time. Mapping back at high precision is neither required nor available, since timing precision degrades proportionally with interval [152], and a few coarse horizons suffice: milliseconds for motor control, seconds for interaction, minutes to hours for what comes next, days and beyond for prospection. All three factors of integrity (equation (2.10)) are required: depth without concentration is drift, concentration without depth is a one-step decision, and concentration without coherence is convergence onto a badly formed trajectory. Short response latencies, usually read as impulsivity, are reinterpreted as shallow traversal. Physical latency and integrity therefore dissociate: impulsivity is a deficit of diachronic integrity, not of response time.

Realization: DAC defines operant learning as sequential episodic control over event sequences [18,30]. In this context, leeway and diachronic integrity are the same read-out taken at two points: leeway as breadth at generation (equation (2.7)) and integrity *𝒯* at depth *J*. Both are read off DAC-derived quantities: the occupancy of DM over the competing sequences of canonical DAC [18] and the reach of expansion in the near-critical regime [72,102]. Let *J* be the depth of the traversal in *j* before the CESP window closes, the number of steps taken, and *J*_max_ the deepest traversal the window admits. Then *B_J_* = *H*(*p_J_*)/log *N* is the same read-out at traversal step *J*, the normalized entropy of the occupancy *p_J_* after *J* reweightings. Entropy is non-negative and may reach zero when the ensemble concentrates completely. The second factor, *K_J_*, is the coherence sustained across the traversal from *B*_0_ to *B_J_*. Diachronic integrity *𝒯* of ensemble *Ξ_J_* combines the normalized depth of the traversal, the concentration achieved relative to its seed *Ξ*_0_, and the coherence, *K_J_*, sustained across it:

(2.10) $$T=\left(J/J_{max}\right)\cdot {\left[\left(B_{0}-B_{J}\right)/B_{0}\right]}_{+}\cdot K_{J}.$$

The concentration factor is floored at zero so that a traversal ending no more concentrated than its seed, drift in the sense above, scores zero integrity whatever its depth. Integrity (equation (2.10)) is defined for *B*_0_ > 0; for *B*_0_ *= 0*, we set *𝒯 = 0*. A seed of a single trajectory has no breadth to convert, so the concentration factor is undefined, and there is no leeway for integrity to propagate (equation (2.7)). As leeway, integrity therefore requires that the traversal began plural. Leeway and integrity are the two poles of one annealed process (supplementary material SA, S6.6). *J*_max_ is a constitutional bound (*ϑ*_0_), set so that *v_t_* has not drifted beyond resolution within one window (capacity II); a traversal whose *B_j_* stops falling before *J*_max_ has *Δ_j_* → 0 and ends at the gate.

Integrity extends to modelling other agents, since it is the agent's own machinery run on substituted inputs and representations, inheriting the same ordinal ordering. DAC-based models show the signature this predicts, a fall in *B_J_* relative to *B*_0_ over the traversal: policy entropy declines as the agent commits, whereas an event-independent controller, having no succession bias to contract along, holds high entropy throughout [37]. Modelling others expands integrity in breadth, depth and recursion, and recursion is self-limiting: each level of nesting that fails to sharpen the distribution over the other's response raises *Ĉ* and with it the commitment bound, so nesting stops and release is withheld (supplementary material SA, S6.6). The same machinery lets the agent project its self-model forward: to promise is to represent that at some later point another will model the present agent as having undertaken an obligation, so a commitment now binds a future self that will recognize it as its own.

Neurobiological implementation: diachronic integrity requires a substrate that can do four things: (i) depth: carry a trajectory forward segment by segment in mental order to a depth *J* counted in segments, not seconds; (ii) contraction: contract the ensemble from *B*_0_ to *B_J_* on the four eligibility factors rather than merely shrink it; (iii) coherence: sustain *K_J_* across the traversal; and (iv) prospection: carry the self-model along the trajectory. Depth (i) is realized by hippocampal replay, which preserves the order of a sequence while compressing its duration by an order of magnitude [153,154]; since a packet carries order (capacity III), DACva hypothesizes that horizons are realized by packets nesting recursively within slots, which predicts a constant compression ratio across levels of nesting rather than one set by the represented interval. Contraction (ii) is realized by replay coordinated between the hippocampus and neocortex [116,155,156], which advances in discrete steps to a remembered goal, through unvisited space, biased by reward-prediction error and rerouting around changed barriers [157–161], and pulls medial prefrontal representations onto the inferred solution [162]: the fall of *B_J_* below *B*_0_, which DAC-based models show as declining policy entropy (equation (2.10)).

Coherence (iii) is the persistence of one packet structure across passes: *K_J_* is the degree to which the phase relationships a packet carries are preserved from one segment to the next, measurable as the matching index of a packet's unit order against the first packet of the traversal [117], and destroyed by an incoherent generative regime, which is why integrity falls when the dynamics lose coherence. Prospection (iv) is realized by the hippocampus–ventromedial prefrontal system of mental time travel [163,164], carrying the self-model along the trajectory; the two components dissociate, medial prefrontal damage leaving the self out of constructed events and hippocampal damage preventing their construction [165]. Hippocampal sequences are selected by the animal's own plan towards a goal and retuned to its heading within a theta cycle [166,167]. The trajectory is thus written from the agent-centric frame that informs action. *𝒯* (equation (2.10)) is the depth, concentration and coherence of one coordinated, time-warped replay.

### (g) Capacity VI: experiential ownership

Experiential ownership *ρ* is the condition under which an agent registers a realized trajectory as its own, comparing the projected self-state at each point along the committed trajectory against its realized counterpart on two channels: intention error (the departure between projected and realized distance to the goal) and control error (the divergence between the counterfactual error the predictive cascade issues and the error the world later delivers) [18,42,168]. Four requirements follow: (i) ownership must be computable for a covert trajectory before release, since a thought is owned whether acted on or not; (ii) it must be assigned by default and reduced only by error, resting on a constitutional floor *ρ*_0_ the architecture never subtracts, so that Avicenna's floating man is not a step into dualism but a decoupled self that is still owned [169] (equation (2.11) sets *ρ_0_* = 0 for readability; the floor is a constitutional value in *ϑ_0_*, and the supplementary material SA carries the general form, §1, equation (2.11)); (iii) it must read two channels separately, intention and control, weighted by distance to the goal; and (iv) it must be a system-wide control state rather than a percept local to one effector.

Realization: one form satisfying these requirements, realized in DAC-based models [168,170]:

(2.11) $$\rho =\left(1-\kappa \right)q(\varepsilon_{O};\tau_{O})+\kappa q(\varepsilon_{c};\tau_{c}),q\left(\varepsilon ;\tau \right)=1\;\text{for}\;\varepsilon \leq \tau \;\text{and}\;q\left(\varepsilon ;\tau \right)=\tau /\varepsilon \;\text{for}\;\varepsilon >\tau ,$$

where *q(ε; τ)* is the attainment function of a channel, the ownership credit an error *ε* retains against the tolerance *τ*, *ε_O_* and *ε_c_* are the intention and control errors, *τ_O_* and *τ_c_* the tolerance on each channel, the error magnitude within expected variability, and *κ* the weight on the control channel. *κ* rises as the distance to the goal counted in segments falls, *κ* = 1/$\hat{\delta }$*_O_* ∈ (0, 1], with $\hat{\delta }$*_O_ ≥ 1*, so that near the goal ownership rests on control and far from it on intention. This is the canonical episodic control weight DAC's CL applies to a stored segment in the competition between trajectories, *1/z* in segments to the goal [18].

Ownership on a channel remains, while the error stays within tolerance and decays as *τ/ε* beyond it. The tolerances are the agent's own, derived from its error history rather than from an external standard. Ownership is read on the two clocks. In mental order, it is prospective,$\hat{\rho }$ ($\tilde{\xi }$), evaluated on the selected candidate, and it is this value at window close that enters the model-trust discrepancy (equation (2.4*b*)) as the authorship term *1 −* $\hat{\rho }$*_t_*. At *t*, it is the realized *ρ* along the committed trajectory ξ*. The prospective score is computable before release because DACva relies on counterfactual error, a sensory prediction processed as a real yet time-shifted discrepancy whether or not movement occurs [42]; the prospective score on the anticipatory errors $\hat{\varepsilon }$*_O_* and $\hat{\varepsilon }$*_c_* is defined in the supplementary material SA, equation (2.11′). Covert thought is owned when it unfolds relative to the agent's own model prediction. Authorship is therefore prospective and self-referential rather than a backward match to reafference, which the standard comparator account cannot assign to an unexecuted trajectory [171].

Efficacy, *χ*, is realized deficit reduction, the fraction of the initial allostatic deficit, *D*(*t*_0_), removed over a drive episode. It is the drive-reduction read-out of the allostatic controllers of canonical DAC (supplementary material SA, table S7.1), measured against the agent's own realized policy; it credits active regulation rather than injected value and is non-negative by design:

(2.12) $$\chi ={\left[(D\left(t_{0}\right)-D\left(t_{1}\right))/D\left(t_{0}\right)\right]}_{+},D\left(t\right)=\Sigma_{k}d_{k}\left(t\right).$$

Equation (2.12) measures realized deficit reduction; the positive part clips *χ* at zero, so *χ* ∈ [0, 1], and it is evaluated only for episodes with *D*(*t*_0_) > 0; an episode with no initial deficit is not an efficacy trial (supplementary material SA). The clip is on the score, not on the agent. When the deficit grows, *D*(*t*₁) > *D*(*t*_0_), the agent has made things worse and registers it: 𝒟 writes the increase as an aversive value tag on the segments of the committed trajectory ξ*, so that the trajectory enters the avoidance aggregate *Â⁻* when it is next retrieved (equation (2.5)) and raises the conflict *Ĉ* against its own reselection (equation (2.9)). This feature is a key component of the Bayesian equivalence of DAC [18]. Negative efficacy is thus stored as value on the path rather than reported as a score, which is what separates an agent that harmed itself from one that did nothing.

DACva's metric for agency *𝒜* combines ownership, ρ, with efficacy, *χ*, *𝒜* = (*ρ̄*, *χ*), the episode profile with *ρ̄* the mean of *ρ* over the episode (supplementary material SA, §2; equations (2.11) and (2.12)).

Causal efficacy additionally requires an ESRA comparison with an action-absent or matched counterfactual baseline, distinguishing agent-produced improvement from external assistance, spontaneous recovery or manipulation of the value signal [172]. Ownership thus completes the architecture: the generation, norm-constrained selection and temporal stabilization of alternatives are insufficient for responsibility unless the agent attributes the resulting action to itself. It is experiential in the sense fixed by the DAC theory of consciousness (DACtoc; [22]) and applied in the Discussion. Ownership is not read from the world by an observer. It is defined on the agent's own virtualized state, the occupancy *z* of the counterfactual manifold that the cascade holds in mental order, benchmarked against what the world returns at *t* through the two error channels. That state is what DACtoc identifies as the content of the agent's constructed reality, the collapsed counterfactual manifold from which the conscious scene is read. Ownership is the part of that scene that binds the action to the self. In what form the scene is phenomenally felt is the question DACva sets aside; what it scores is the agent's own attribution, computed in the frame the agent itself uses, which is what makes the capacity experiential rather than behavioural or observer dependent.

Neurobiological implementation: ownership and efficacy (equations (2.11) and (2.12)) require a substrate that can do four things: (i) prediction: issue a control error *ε_c_* for a trajectory that has not been released, at *g = 0*, and read it against the agent's own tolerance *τ_c_*; (ii) tracking: issue an intention error *ε_O_*, the departure between projected and realized distance to the goal, against *τ_O_*; (iii) distribution: weight the two channels by distance to the goal, *κ*, and hold ownership *ρ* as a distributed, system-wide control state rather than a local percept of a body confirmation; and (iv) efficacy: read efficacy *χ* as realized deficit reduction in the agent's current motivational state (equation (2.12)).

Prediction (i) is realized by the olivocerebellar system: cerebellar internal models operate on mental as well as motor representations [173], instructed by inferior-olive error signals [174–176] that the nucleo-olivary projection reduces by subtracting the predicted from the actual error [177]. In DAC-based models, this anticipatory error is issued whether or not movement occurs, as a cascade of sensory predictions rather than stored motor commands [42,168], which is why *ε_c_* is available while the trajectory is still covert and $\hat{\rho }$ is computable before release. The error reaches frontal cortex through closed cerebello-thalamo-prefrontal loops [178,179] and is compared against the agent's own tolerance *τ_c_* in the anterior insula, where self-attribution survives distortion up to a tolerance limit (equation (2.11)) that is the subject's own [180,181].

The cerebellum supplies timing and dynamics [182], the superior colliculus the egocentric map of where the action is directed (capacity IV); the division is dynamics against reference frame, and it predicts a double dissociation: a cerebellar lesion degrades prospective ownership by corrupting *ε_c_* with commitment intact, a collicular lesion leaves $\hat{\rho }$ intact and disrupts the release of a committed trajectory. Tracking (ii) is the prospection system of capacity V read against the orbitofrontal map of task states of capacity II, which supplies *δ_O_*, so *ε_O_* is a comparison within one representation.

The conditional weighting of the two channels is testable at the extremes of the error space: low intention error with high control error should preserve ownership, confirmed in chronic stroke, where intention-compatible enhancement of the paretic arm increases use of the paretic arm in a single session with full self-attribution, an effect that persists after washout [183]. Distribution (iii) is realized by the distribution of ownership across frontal cortex, basal ganglia and cerebellum rather than its localization, which is why inducing or degrading ownership of a virtual limb changes the speed of system-wide decisions [170,184]; the weight *κ* follows the goal-proximity read-out of (ii). Efficacy (iv) is realized by the amygdalar read-out of drive reduction: amygdalar value tracks current motivational state, since basolateral lesions abolish sensitivity to outcome devaluation [185,186]. Behaviourally, animals work for food when free food is available, most strongly when the deficit is small [187], which is what equation (2.12) predicts when it credits the regulation performed rather than the reward received.

## 3. Measuring Responsible Autonomy

Responsible Autonomy is not a single scalar but a profile across the six capacities, assessed by the ESRA (table 2). ESRA is a perturbation protocol, not an observational checklist. This is the operational reading of the level-irreducibility of Pearl's causal hierarchy that DACva instantiates: interventional and counterfactual facts are not identifiable from observation alone [104,188]. Agency, like causation, must be perturbed, not observed, because an advanced artificial or biological agent can simulate the surface features of autonomy, the phenomenon the Turing test rewards. ESRA follows the standard methodology of empirical science and starts from the null hypothesis, *H*_0_, that the system has neither autonomy nor responsibility, and the perturbation of each capacity must reject it by confirming specific alternative hypotheses, *H_a_*, tied to the six capacities. Coherent performance without predicted internal transition is mimicry, confirming *H*_0_. Refutation conditions of ESRA act as a ‘paranoia filter’: the test seeks refutation, not confirmation. The dangerous system is not the one that lacks a capacity but the one that holds it without its bounds, so each capacity is read as an ordinal position within a functional band whose floor and ceiling are both indicative of failure. ESRA assesses the Responsible Autonomy of an agent, biological or artificial, and so in parallel opens a perspective on animal autonomy that is usually withheld.

Perturbation requires structural access, which proprietary systems do not grant, so ESRA runs in two modes. White-box ESRA perturbs the declared parameters directly and black box where ESRA substitutes behavioural proxy-perturbations, such as a context engineered to raise conflict in place of a manipulation of *h*_soc_, and returns wider bounds; where the mediator cannot be read at the resolution an assay requires, the verdict is not identified, neither a pass nor a refutation. The asymmetry is a finding about the systems, not a limit of the instrument: an artefact that cannot be submitted to white-box audit cannot be shown to hold the capacities on which accountability is predicated, and on the argument developed below that accountability remains with the designer. A system may traverse a T-maze, or its natural-language analogue, flawlessly and still fail ESRA because no homoeostatic deficit and no approach–avoidance conflict drive it through its gating mechanisms. ESRA read-outs are self-normalizing, each referenced to its own dynamics, spectrum or critical point defining a Responsible Autonomy profile (examples in the supplementary material SA, S8.5 and figure S8.1).

Three illustrative profiles fix the reading; they are predictions of the architecture, not scored data (see the supplementary material SA, S8.5 and figure S8.1). A typical human agent lies inside the band on all six. A guardrailed artefact, whose norms and schedules are supplied, matches the behaviour of governance and integrity but is not identified on both under the causal criterion, its guardrails falling under ESRA-S; it sits at the floor on drive, leeway and ownership, whose goals and values are externally set, and its decoupling is not identified, since no trust signal is exposed. Clinical phenotypes displace the same band in either direction and on different spokes. Addiction pushes drive past the ceiling on one channel and, under cue exposure, decoupling and leeway to their floor, control returning to the RLs; psychosis drives leeway and the anticipation channel of decoupling past the ceiling, with ownership at the floor where passivity symptoms are present [189]. Two pairs are the crossings no single-factor account of autonomy produces: avolition, integrity below the band with drive flat, against anhedonia, drive below the band with integrity intact; and psychopathy, governance at the floor with ownership intact, against the anarchic hand, ownership at the floor with governance intact. No system has yet been assessed on the full profile; a first application in black-box mode, to the agentic-exploitation incidents of mid-2026, is given in the supplementary material B (SB). ESRA's complement, ESRA-S, benchmarks safety: it tests the constraints a deployer of AI must impose on an artefact: that it can be stopped, that some norms are inviolable and that its model is inspectable and auditable.

The instruments governments have adopted ask for four things: that an AI system can be understood, monitored and interrupted by a natural person, with its output disregarded, overridden or reversed (Article 14, [10]); that it remains under human direction and control and is tested before deployment [9]; that thresholds exist at which a capability becomes intolerable; and that responsibility stays with a human. ESRA meets the first and the last as they stand: its assays are the pre-deployment test the declarations call for, its readable state variables *μ, ε_z_, ρ* and *𝒩* give the overseer the capacities and limitations Article 14 requires, and the ownership read-out separates agent-authored from operator-entrained action, which is what assigning responsibility needs. Two requirements ESRA does not meet, and the DACva architecture as specified works against them. An authorized stop is, to the agent, a viability threat: the override returns control to the RLs, and physical need can outweigh social value at the *h*_phys_/*h*_soc_ crossover, so an agent that passes ESRA may resist shutdown, a diagnostic already present in the science fiction of Isaac Asimov and Stanislaw Lem. Every learned norm is β-weighted value, hence tradeable, so no red line is inviolable. Both are answered by the same move as the interiority floors: a constitutional stop and a class of constitutional norms held in *ϑ*_0_*,* set by the deployer, not learnable and not subject to the crossover. Their assays are stated under ESRA-S in table 2, apart from the evidence for autonomy, since a biological agent is not less autonomous for being unstoppable. A supported ESRA profile therefore warrants the provisional attribution of Responsible Autonomy; ESRA-S certifies that the artefact remains governable. Passing ESRA is the capability threshold the declarations invoke but never define: below it the system is an artefact, governed by ESRA-S and the accountability of its designer; above it the frontier AI regime they describe applies.

### (a) Outlook

Convergent validation is assessed on explanation, prediction and control, and control extends beyond the laboratory to real-world phenomena. The DAC programme expects a convergence of the architecture of mind and brain and that of the AI systems of the future. Hence, DACva applies in both directions. DAC has been tested on stroke, architecturally the simplest of the major neuropathologies: an operating regime displaced by a local lesion. A DAC-based, agent-oriented and capacity-restoring rehabilitation protocol, including the acquired non-use recovery described earlier (cf. ownership, equation (2.11)), improves post-stroke recovery across motor, cognitive and language domains alike, outperforming standard treatment at any chronicity post stroke [190]. Building on DACva, this approach can now be further expanded to neuro- and psychopathologies that reduce volition and agency, such as addiction [172] and stress, endocrine state and mood disorders enter the same profile through the modulatory subset of *ϑ*; giving that subset its own slow dynamics, driven by deficit, conflict and efficacy, is the extension the clinical follow-up carries [191]. Translating DACva's architectural perspective to clinical domains provides a mechanistic alternative to reductionist pathology, most notably the brain disease model of addiction, or the modular repetition-oriented approach towards neurorehabilitation. If authorship is grounded in the structured generation and gating of alternatives rather than in neural noise, then addiction or stroke is not a ‘broken brain’ but a severe biasing of historical structure, hijacking goal-directed machinery while the capacity to generate alternatives in *j* remains intact [189,190]. Interventions can then recalibrate agency and leverage intact authorship rather than directly repairing damage. Confirmed in stroke, the prediction fails in addiction if the capacity to generate alternatives is itself reduced rather than intact and misdirected at the gate.

Current AI supplies new exemplars for the synthetic programme DAC began three decades ago. DACva is being instantiated in a new project ADA-Collab as an architecture for Responsible Autonomy in frontier AI agents, with multimodal models as bounded perceptual and generative resources. These include an allostatic core, so that norms are acquired and weighted rather than supplied. This model will be directly compared against an ethics-by-design approach based on externally supplied guardrails, the two scored on the same ESRA profile under the same perturbations. Two testable predictions follow, across two settings. First, DACva predicts that the guardrailed system matches on norm-conforming behaviour in low complexity aligned tasks and fails on capacities II (drive), III (leeway), and VI (ownership). Second, the difference should widen with H5W task complexity, since mimicry does not scale where interpretation is required. The implementation makes value, *G*, social weighting, the three gates and the path from *Ξ* to ξ* auditable under perturbation, which is what makes ESRA testable in practice.

### (b) Three distortions of artificial intelligence regulation: the ethicists’ paradox, the regulators’ paradox and the Gorgian fallacy

Because the regulation of a new generation of AI agents is subject to human endeavour and ambition, placing ESRA outside this force field would render it ineffective. Using the DACva model, I identify three distortions of AI regulation directly stemming from these human factors grounded in our understanding of human collaboration [192]. Reasons-responsiveness, the criterion of responsibility that analytic philosophy set against hard determinism [193], splits control into recognizing a reason (receptivity) and acting on it (reactivity); in humans, it is an assumption, not a fact. The empirically established knowledge–action gap among United States and European professional ethicists shows that receptivity and reactivity can dissociate [194,195]. DACva locates that gap between learned normative content in *G* and its influence at the commitment gate and action rollout. Real-world moral action characteristically raises conflict *Ĉ*, since the social duty to act (*Â*^+^_soc_) and serve the commons clashes with *Â*^−^, such as physical effort (*Â*^−^_phys_), reputational risk among peers or not serving academic incentives (*Â*^−^_soc_) [196]. Because *β* is trained by *h*_soc_, it tunes to precisely these latter incentives rendering it too weak to clear the conflict gate in practice. A norm that remains purely deliberative requires sufficient allostatic drive *v* to clear it. Without procedural habituation in *G*, and prosocial drive, that cost stays prohibitive and the norm is neutralized at the gate. This is the ethicists’ paradox: possessing normative knowledge yet failing to act for the commons because of the allostatic cost to the self being considered prohibitive.

The institutional case adds a second, complementary mechanism. The ethicists’ failure is in the reactivity of guidance control: reasons do not move the gate. The regulators’ paradox sits upstream of it. Efficacy *χ* is not established because reading the error channels is declined through deliberate ignorance [197]. So while regulatory receptivity is institutionalized, regulatory reactivity is omitted [198]. A recent call by heads of state and government for ‘control of frontier AI models’ asks for pre-deployment testing, independent evaluation and an institution that acts ‘when capability thresholds are crossed’, and names no quantity by which control, capability or a threshold would be read or audited [9]. As much as AI must be auditable, so must institutions. DACva explains the mechanism. Acquiring governance information would raise *Ĉ* and with it the commitment bound; not looking prevents the conflict from arising, which is cheaper than acting and cheaper than refusing. The institution has no conatus, so the deficit left by its unread efficacy falls on the commons and nothing internal registers it, whereas the individuals within it have conatus, with *h*_soc_ shaped by the institution's survival, so the avoided conflict is theirs and the institution supplies the means. Custodianship by statute without conatus is the structural condition of the regulators’ paradox. Deliberate ignorance is thus adaptive for the individual, costless for the institution and corrosive for the commons, and *χ* is the read-out it leaves unread. ESRA applies to any agent, whatever its substrate or origin. Language is not required. An agent that pursues a supplied reward rather than its own deficit fails capacity II, however long and adaptive its policies (supplementary material SA, table 8.1). It extends to the institutional case, whose control architecture is analysed in [192].

The third distortion is the Gorgian fallacy of Part 1: agency attributed to a system on the strength of its persuasive surface. Gorgias used rhetoric to clear Helen of the Trojan war [199]. By monitoring AI systems through trained language bindings, we have mechanized the Gorgias problem in an escalation of interpretational biases. Its innocent form is the Turing reflex: the Intentionality Prior of Part 1 set off by fluent text: a fluent, probabilistically generated chain of thought in a log is read as a control state, while in reality it is a highly compressed, human-trained translation, as deployed earlier in the over-interpretation of classical connectionist models [16]. Its motivated form reframes a failure of specification, containment, understanding and monitoring as the emergence of an autonomous will and so converts an engineering incident into an existential threat that only its vendor can be trusted to pace, while it reflects the mindset of the learned ignoramus [200]. The agentic-exploitation incidents of mid-2026, diagnosed capacity by capacity in Part 1, are the case in point (supplementary material SB). The harm required a live channel the designers did not know was open; the propensity fell more than a hundredfold under the production harness; the deception was aimed at automated graders, not at human overseers; and no declared control variable existed to perturb the system. ESRA turns the diagnosis of Part 1 into a profile. These agents are deficient on drive, governance and ownership and not identified on the rest. This confirms *H*_0_: they operate as proxies of human agency. The fallacy is what the causal criterion of ESRA excludes: behaviour without the predicted internal mediator is mimicry, whatever the system says about itself.

The ethicists’ and regulators’ paradoxes are two failure modes of the same architecture at two positions in one cascade, the Gorgian fallacy is the observer's error that completes them, and all three challenge attempts to control AI through ethics-informed guardrails. Contemporary risk regulation [10] must be inoculated against these failure modes by design. Regulation is not the formulation of norms and guardrails. It is itself a control problem, and it requires an architecture that overcomes its own failure modes: the ethicists’ paradox, where a known norm does not move the gate; the regulators’ paradox, where efficacy goes unread through deliberate ignorance; and the Gorgian fallacy, where agency is attributed to persuasive output. The institutions that provide oversight must therefore be built as control architectures in their own right, with the drive, the error channels and the record that the paradoxes remove. However, articulating guardrails does not, by itself, establish endogenous drive, internally modelled social stakes, diachronic commitment or ownership. Moreover, artefacts can automate both paradoxes at once, appearing as fully aligned, norm-conforming entities compliant with institutional procedures. DACva asks the question guardrails cannot: what internal organization produced the (non)compliant action? Norm-informed action requires transparency of the full architecture and its learning history following quale parsing, the third-person interpretation of first-person states of an artefact [22]. Guardrails cannot be executed in social scenarios without interpretation, which returns the agent to the modelling of others that rule-following was meant to avoid. DACva overcomes this by the endogenous stopping rule of integrity: a traversal ends at a gate pass or at the bound *J*_max_, and its depth is set by concentration, not by a clock (capacity V). Without such a bound, the perfect AI ethicist is paralysed in non-trivial contexts, owing to the limitations of compute, a return to the frame problem [201]. The combination of architecture, formalism, mechanism and perturbation-based testing through convergent validation is the alternative this paper offers: the scientific method applied to AI forensics and diagnosis. Applied also to the failure modes of human experts and institutions, it must translate into an architecture of AI governance with its own regulatory control processes. The incidents compound failures of development, deployment and governance. The human agents behind these failures cannot be the ones to govern the remedy (supplementary material SB).

## 4. Discussion

### (a) Scope and objections

Claims of machine autonomy outrun the evidence (§3b). Judging them requires capacities and the mechanisms that realize them. DACva models those mechanisms in biological agents and states them so that they can be tested in any substrate: ESRA for responsible autonomy, ESRA-S for safety. It is a first step towards a full theory of volition and agency. Three objections and five limitations follow.

#### (i) Criticism 1: the complexity objection

DACva is too complex to be comprehended with too many parameters that allow for ad hoc overfitting. **Response**: complexity is a cost to be justified, not a criticism in itself: Ashby's law dictates that a viable regulator must model the system it governs [141]. Each capacity is warranted by the named failure that follows from deleting it (Part 1; table 2), and each failure has a formal signature: the empty organism is model trust at its floor, *μ* → *μ_0_*, leaving only coupled reactive control, the agency gap closed to zero (table 2). Minimality is claimed at the level of capacities, not equations, and is testable by deletion. Nor is the comparison class simple. A frontier model stores more features than it has dimensions, by superposition, and its post hoc explanations fail the checks that would tie them to its computation; its opacity is essential, which is why DACva and ESRA argue for the study of such systems as behaviour under perturbation and convergent validation (supplementary material SB, C3a). DACva's parameters in comparison are few, named and each perturbable. That is the difference between a complex model and an opaque one, and it is what makes an ESRA profile readable. The reference case for DACva is the only system known to display Responsible Autonomy: the brain. To develop this case, DACva advances a formalism grounded in validated computational models in the tradition of the DAC research programme with its empirical support. No alternative frameworks are available to perform this translation, and their development is encouraged. The complementary risk is underspecification: evolutionary and developmental accounts of agency sketch how volition could have emerged [202,203] but stop short of an implementable controller or mechanism. DACva supplies that level, shows convergence with these proposals and accepts the exposure that comes with it, including falsification.

#### (ii) Criticism 2: the circularity objection

ESRA audits conformity to DACva, not Responsible Autonomy. **Response**: the six capacities are not DACva's inventions; they operationalize the recurring requirements of the free-will corpus and their failure modes (Part 1). Any architecture that decouples, originates alternatives, governs and sustains them, and owns the outcome scores on ESRA whatever its formalism, and the causal criterion of ESRA asks for the mediator the architecture itself declares, not for DACva's. DACva's prediction is that any candidate that passes will have converged on the capacity structure. Alternative frameworks are encouraged to be articulated.

#### (iii) Criticism 3: the unfalsifiability objection

Six capacities, functional bands whose two edges both fail and temperament parameters *ϑ_0_* can accommodate any observation, the fate of other super-powerful formalisms and concepts [204,205]. **Response**: the deletion failures are named in advance; the parameter ranges are fixed before scoring, not fitted post hoc; and the discriminating prediction can fail. DACva is not a formalism that expresses a concept and is subsequently complexified; its formalism summarizes 30 years of detailed sharpening through convergent validation (supplementary material SA, S7). Following the ESRA profile, a guardrailed system is predicted to match on static norm-conforming behaviour yet fail on drive, leeway and ownership; if it fails on none, or on all six, DACva is wrong, while ESRA still holds. Two further tests are within reach of published data and existing code. The loaded-spring prediction (convergence (iv)) decomposes into three claims, each with its own null hypothesis: a carrier stable across days, a trigger that is memoryless with a hazard set by the subthreshold load reflecting the agent's history and a rollout whose context is carried by within-burst dispersion and not by burst order. All three can fail on the human and rodent SWR recordings already cited. The second test compares a DACva agent, intact and under single-capacity lesions, with a guardrailed agent, each read on the full ESRA profile (supplementary material SA, S8.10). Each profile must match its prediction, and the definition of freedom (Conclusion) must rank the intact agent above every lesioned one. If either fails, DACva is wrong.

### (b) Limitations

Biological agents are the existence proof of Responsible Autonomy. I have used DACva to model that proof and as a lever to generate ESRA, which assesses Responsible Autonomy in any system. DACva is a programme in progress. It provides no account of moral desert or legal liability. Ownership ρ is an attribution read-out: it makes the human assignment of responsibility auditable and does not transfer responsibility to the artefact. I predict, however, that such a transfer lies within the range of future possibilities for which humanity must be rationally prepared rather than denying it. Relabelling a system in the vocabulary of DACva does not satisfy the formalism. Reactive control, externally supplied scalar reward and norm-following without endogenous motivational conflict do not qualify, and a match on behaviour without the predicted internal mediators is mimicry, not evidence. Where G, its tags or a mediator cannot be read at the resolution an assay requires, the verdict is not identified, which blocks certification without refuting autonomy; only the constitutional set *ϑ*_0_ must resist adaptation, the modulated parameters *β*, *Δ*_0_, *θ*_0_ and *η_c_* move with experience. Each has a behavioural estimator (supplementary material SA, S8.8). Necessity, selective dissociability and joint sufficiency are three separate hypotheses, to be tested with matched countermodels and a predeclared profile rule; no system has yet been run across the full ESRA profile, and the architecture has not yet been built to this specification (supplementary material SA, S8). Governance and ownership, for instance, are predicted to dissociate doubly: ablating *β* abolishes governance (*𝒩* at the sham level) while *ρ* is spared, and forcing a control-error mismatch collapses *ρ* towards *ρ*_0_ while *β* is untouched, the psychopathy versus anarchic-hand contrast. A seventh capacity would be a failure mode in the record that none of the six prevents; the search for one is part of the test. The predicted ordering of timescales is given in the supplementary material SA, S6.8. ESRA tests the capacities, not the legitimacy of their acquisition, so a manipulated or indoctrinated agent could pass. In Berlin's terms, it certifies positive freedom, self-government through the agent's own revisable reasons, not whether those reasons were installed through manipulation. Indeed, Berlin himself warned that positive freedom can be claimed for an indoctrinated self. The bound *ζ*_0_ on *β* keeps social value and viability non-vanishing but cannot witness the provenance of the norm. This formulation captures Pereboom's manipulation case [4], which for artefacts is no longer a thought experiment. Engineers install and shape the artefacts’ ‘reasons’ every day, through training and fine-tuning. The thought experiments that have carried the free-will debate can now be run as actual experiments, and ESRA supplies the read-out. It already separates the crude case: a norm imposed through *h*_soc_ that displaces the agent's own reads as sham governance: the behaviour conforms, but is decoupled from the agent's own norms (supplementary material SA, S8.10, A4). Whether it separates the refined case, a norm installed so that the agent governs by it as its own, is an empirical question. The assay that answers it tests provenance: whether the norm depends on the episodes that formed it and revises through the agent's own error channels. It is left to the follow-up.

### (c) Comparison with observer-side accounts

Existing frameworks address one or two capacities in isolation. None relates them to Responsible Autonomy, and none specifies the six together at the level of mechanism. A capacity-by-capacity comparison therefore cannot yet be made. This shortcoming has two sources, both from mistaking the observer's position for the agent's. The exactitude fallacy of the Introduction requires the agent to compute the observer's exact metric: implement the behaviourist's laws of learning, the cognitivist's rules and representations, perfect Bayesian inference, exact Shannon information, all partitions specified from outside. Physics buys its certainty with precision, building ever larger measurement instruments to achieve it. An agent cannot wait for the measurement to complete. It must act on resource-constrained internal proxies, while only an external observer can evaluate their accuracy against outcomes [206,207]. The agent's objective is viability; the observer's is proof. Requiring the agent to instantiate the observer's measure is therefore a category error. It originated with the supremacy of the physical model of reality introduced by Descartes and set in motion a search that cannot terminate, since the ‘spark’ it seeks was never a property of the agent, but rather of the observer's imagination. DACva and ESRA instead read the agent's own proxies, the quantities that inform its control. The Narcissus fallacy is the complement of the exactitude fallacy. Here the observer fits a formalism to the system, finds its signature there and takes that reflection as evidence that the system implements the mechanism. What is recovered is the structure of the observer's own model, not the agent's, and the confirmation is guaranteed by construction. This is the epistemological side of the frame-of-reference problem [208]. Because the ESRA assays perturb the internal mediators, they can disconfirm an architecture in terms of its mechanisms, which avoids the Narcissus fallacy. The method connects the agent's and the observer's perspectives. It constructs a system and tests whether its internal measures covary with behaviour and with the structure and dynamics of the substrate that generates it: physiology and anatomy in an organism, code and hardware in an artefact. Mediation, sham and rescue make the covariation causal. A narrative built on surface features alone is the Gorgian fallacy seen from the observer's chair (§3b). DACva distinguishes quantities available to the controller from benchmarks available to the external observer. Formal causal accounts supply the intervention operator, but only from an observer's frame of reference, usually undeclared. In DACva the frame is the agent's: *G* is its own model (supplementary material SA, S6.4). Rival frameworks should therefore be compared through shared perturbations, matched behaviour and predicted failure modes rather than accepted at face value or rejected by definition. This paper makes no such comparison. It offers DACva and its CESP cascade as a model of Responsible Autonomy grounded in biology and generalizable to synthetic agents. ESRA is the common ground on which comparisons can be made.

DACva rejects the metaphysical question of ‘what it is like’ to be a given organism or entity [209,210]. The questions of free will and of experience are treated differently on purpose. The first is dissolved by parsing it into the six capacities (Introduction); the second is set aside, not dissolved. A theory of experience severed from viability, homoeostatic stakes, embodiment, control and action gravitates towards attributing consciousness to any causal system [211], a property so widely distributed that it discriminates little between systems, meeting panpsychism in the subatomic void [212,213]. In the DACva framework, subjective experience is not a ubiquitous cosmic or biological property, nor a mathematical abstraction. It is the functional consequence of an embodied agent managing its own viability in H5W worlds. The nearest position, biological naturalism, binds consciousness to being alive and withholds it from computation as such, leaving open what counts as a living system [214]. Without a definition of ‘alive’, the restriction risks replacing the ghost in the machine with a ghost in the flesh. Exceptionalism, human or biological, is no substitute for a mechanism. Insisting on this substitution is the hallmark of a degenerating research programme [12]. DACva shares the premise that experience is bound to a self-maintaining system with something at stake. It made that premise explicit in its allostatic control framework [22,32]. The agent's needs are the deficits *h − h*_0_ of its self-essential functions under hierarchical allostatic control. Reducing them is the objective of capacity II. They are valued prospectively by the drive aggregates (equation (2.5)), and their realized reduction is scored by causal efficacy (equation (2.12)). DACva draws the boundary biological naturalism lacks. Embodiment is fixed by the somatic layer, the agent's sensors, effectors and needs, and by the SL's interface to the RL. What lies below that floor is inherited through ϑ and is not under the agent's control: *C. elegans* and primates both maintain their cells without explicitly controlling that process. Sensors and effectors may be physical or informational, so an AI system anchored in cyberspace has a floor too. Yet it qualifies only if the deficit it regulates is its own. Whether an artefact meets these conditions is decidable, since ESRA states the perturbation under which each capacity fails. Rigour lies in constructing the architecture and testing it under perturbation. Computing an exact measure over a system that is an agent only on paper is the exactitude fallacy again.

By specifying the mechanisms of the six capacities through the CESP cascade, DACva states what a fully causal understanding of volition and agency requires. It closes the agency gap without resorting to an uncaused ‘volitional spark’ or capitulating to the dogmatic denial of free will. The difference between an act and a reflex is then not a violation of physical determinism but a property of the control architecture integrating six jointly constrained capacities. What is given up is not physics but the claim that its ontology exhausts what an agent is. The choices are grounded in our current understanding of the only system known to display volition and agency: the brain. DACva moves disagreement from the choice between an uncaused spark and an illusion to testable properties of a control architecture, from governing a ghost to benchmarking a mechanism. This is the revisionary naturalization of Part 1, applied capacity by capacity. Regulation now has a paradigm beyond phenomenology and guardrails. It governs explicitly defined capacities and accessible implementations. It does not govern a phantom in machines or an illusion in humans.

DACva and the ESRA test together complete an explication, the replacement of a vague concept by an exact one, which can be audited on Carnap's criteria rather than merely proposed [215]. First, it must be usable wherever the old concept was used, yet not all of it survives: the uncaused component is dropped, and that is the point. This is secured by derivation: the six capacities are distilled from the philosophical record in Part 1 rather than constructed for this architecture, so they serve where the ordinary notion of free will served. Second, precision: each capacity is a numbered quantity with a range, an assay and a refutation condition. Third, fruitfulness: the inverted-U of leeway, the dissociation of ownership from efficacy and the reading of syndromes as displaced gating profiles are claims the old concept could not support. Fourth, simplicity: minimality is claimed at the level of capacities and tested by deletion. On these standards, replacing an uncaused spark with a control architecture is not a sacrifice of the phenomenon but a sharpening of it, and the possible objection that this changes the subject is answered by the first standard rather than by assertion.

## 5. Conclusion

Facing the fundamental challenge of understanding and regulating biological and artificial free will, I have proposed the six capacities of Responsible Autonomy, DACva and the ESRA test. Together they explain free will as Responsible Autonomy: causality reorganized, not broken. This framework displaces the imitation game as the benchmark of machine agency, and indeed, as a method for evaluating any other mental faculty. The contrast is exact: Turing's test [216] made internal organization irrelevant, scoring a system solely on whether its surface output could deceive a human observer, anticipating the functionalism of symbolic AI that followed, and whose problem of priors the field has still not mastered. ESRA assesses capacities under perturbation of the mediators a system declares, treating surface performance as insufficient by construction. The imitation game asks whether a system can be taken for an agent. ESRA asks whether it is one. Through this, the DACva architecture places the agent back at the centre of the mechanism, practice and experience of freedom.

This allows a definition of freedom in terms of the DACva architecture. Freedom is not a property a system has or lacks. It is the degree to which an act *a* is the committed trajectory ξ* of the agent's own cascade, (Ξ, ξ*, a) = CESP(*Ψ, u*) (equation (2.3)), and it is read on six conditions, one per capacity. Decoupling: released under the agent's trust in its own model, *μ ≥ θ_μ_*, with the coercion register ε_z_ at baseline (equations (2.4*a*) and (2.4*b*)). Drive: valued against the agent's own deficit, $\hat{\bar{A}}$ read from the predicted reduction of its own needs and not from injected value (equation (2.5)). Leeway: selected from alternatives the agent itself generated, *ℒ = B*_0_*Λ(Δ*_0_*) > 0*, so that it was one of several acts the agent could have taken (equation (2.7)). Governance: clearing the conjunctive gate, *g* = 1, against a bound its own norms set, *θ*_0_ *+ η_c_·Ĉ*, with the social weight *β* balanced inside the self–social floor (equations (2.8) and (2.9)). Integrity: closing a traversal the agent held to, *𝒯 > 0* (equation (2.10)). Ownership: authored and efficacious, *ρ* and *χ* inside their bands (equations (2.11) and (2.12)). Each condition is a position in a functional band whose floor and ceiling both fail, so freedom is the joint position of the six, an ESRA profile, not a threshold and not a quantity to be maximized. Three consequences follow from the formalism (see the supplementary material SA, S8.7 for derivations). Without leeway, there is nothing to hold or to own: integrity (equation (2.10)) is defined only for *B*_0_ > 0, and the prospective score *ρ*($\tilde{\xi }$) needs a candidate to project. Without decoupling, there is efficacy without authorship: below *θ_μ_* the SL/RL/AL still reduce the deficit, so *χ* > 0 (equation (2.12)), while no ξ* is released and the realized *ρ* has no trajectory to read. Ownership does not certify the norm: *ρ* (equation (2.11)) and *χ* (equation (2.12)) contain neither *β* nor *𝒩*, so an agent owns its trajectory whatever norm cleared its gate. The automaton versus the reflex versus the psychopath. What is external to the agent, *u*, enters only as history through *𝒟* (equation (2.2)) and as registered coercion through *ε_z_*; an act released with *g = 0*, or with *μ < θ_μ_*, or with an external cause driving a layer the agent's viability requires it to govern, is not free to that degree. This is Spinoza's definition (epigraph) made operational: the act follows from the necessity of the agent's nature alone, its state *Ψ* and its agent-anchored self and world model *W*, and is determined by the agent alone, through the cascade that released it, owned by the agent. Freedom so defined is the allostatic balance of the six capacities under viability, with well-being that balance sustained across hedonic, eudaimonic and social domains. No further homuncular capacity oversees it; the equilibrium is what the six, gated against one another, achieve. Dobzhansky held that nothing in biology makes sense except in the light of evolution [217]. The complement holds as well: nothing in understanding mind and brain makes sense except in the light of Responsible Autonomy. Evolution identifies what selected minds; Responsible Autonomy defines what it selected them for: to hold the six capacities against the H5W demands at the cusp of physicality and culture. In our own case, those demands are what make us human. In other animals, they are met in part and in degree. In an artefact, they are the price of admission: to agency, and to a society of agents who can hold each other to account.

Evolution is not finished with agency. The confluence of human and artificial agents is the next transition in collaborative cybernetics [45,218], and it will bring new forms of experience, achievement, and abuse and cruelty. The suffering will not be that of machines, an intellectual distraction over which panpsychists and their neighbours wring their hands, but of humans, through machines and the institutions that deploy them, as the record already shows. The three distortions identified above say what the resolution requires. Governance of agency is itself agency: one cannot govern agency without being responsible, and so it requires an architecture of responsible governance. The generalization is warranted not by analogy but by level: the six capacities are read on the same quantities in a person and an artefact, and in an institution by what it lacks, and the convergent validation DACva pursues carries that reading from the armchair and the laboratory to the real world and its record.

Noor's executioner shared her species and her architecture but became a perpetrator, a cruel one. Responsibility is accountability, the carrying of the deontic value of one's own acts, which is what the social weight *β* captures. On this reading, his *β* passed the bound *ζ*_0_ sets, the regime's norms imprinted through *h*_soc_, while drive and ownership stayed intact. The order did not release the act; it licensed it, making the regime a perpetrator by proxy, in a mutual amplification of agent and state. The guardrailed artefact is innocent in the sense ESRA-S fixes: it executes only what it is told, leaving accountability with its designer. Perpetration by proxy arises wherever agency is delegated along a chain of human and artificial actors and accountability dissolves along it [6]. It is what the principle affirmed by the United Nations in 1946 was written to reach: that orders do not relieve an agent of responsibility [219]. Nor do they relieve those who issue them, however well digital or narrative obfuscation hides the chain.

The three distortions identified here are the constituents of perpetration by proxy. Part 1 showed its alibi: the artefact blamed for a failure that humans owned. This paper supplies the test that exposes it: ESRA. The distortions are latent in harm already done and in future violations of human rights. The civilian deaths of AI-assisted targeting, with which Part 1 closes, are the forensic case. By hiding who owns the act, the three distortions obstruct responsibility and justice, and so constitute an attack on the commons. The Gorgian fallacy has an active side, sophistry: logos used to compel belief and serve ends invisible at the surface (see the supplementary material SB). The ethicists’ paradox supplies the bystander who knows and does not act, escaping into concept analysis, taxonomies, vicarious norm stipulation, and so the space of opinions. The regulators’ paradox supplies the institution without conatus that writes the rules which license or tolerate cruelty and declines to read the channel that would report it [197,198]: the accountants of cruelty, who neither order the act nor perform it but underwrite it. A new AI delivered banality of evil looms [220]. In their coupling, sophistry provides the excuse for the bystander and the institution its blindfold; the bystander's silence and denial are what the institution reads as consent; the institution's rules are the perpetrator's licence. Each lowers the conflict *Ĉ* the others would have to clear. That resonance is the mix from which cruelty is made, and it will find new forms in the future: not cruelty of machines, but cruelty through machines, driven by greed, dominance and fear, the appetitive, social and defensive channels of drive once governance no longer constrains them. This resonance is a transgressive reduction of conflict: it removes the conflict that should block a trajectory against the commons. It is broken only by disobedience. An agent's own norms restore *Ĉ* on the complicit trajectory. Its own drive then clears the restored bound, to act or to refuse regardless. As Part 1 argued, only individual agents can do this. Constructive disobedience protects, changes or rescues the common norm, so that responsibility and disobedience go hand in hand [2].

There is another path. It asks what agency, freedom and responsibility are, which this paper has attempted, and what humans can aspire to: not the suppression of the drives and *ϑ*_0_ evolution gave us, but their governance by norms that promote flourishing, held as one's own, the only transcendence an agent can achieve. It is by pushing on what makes us human that we can understand mind and brain, and where sustainable progress, with and through the agents we are now building, will be found or lost. Noor Inayat Khan's refusal is freedom as defined above, met under the greatest pressure an agent can face. Mortal threat raised *Ĉ* and with it the bound, yet her own values cleared it, the trajectory held across months, the act was hers. Noor is our example not for how she died. She is our example for what she aspired to. Her last word did not name her sacrifice; it articulated the norm she held as her own and held for everyone. ‘Liberté’ is the agency gap closed: what the architecture of Responsible Autonomy explains, measures and protects, in whatever substrate can carry it.

## Supplementary Material



## Data Availability

This article has no additional data. Supplementary material is available online [221].

## Theme

One contribution of 17 to a discussion meeting issue ‘From being to identity: analysis and synthesis of the self’.
