# Supplementary material for: The architecture of Responsible Autonomy in biological and artificial systems: the Distributed Adaptive Control of volition and agency
Source: Philos Trans R Soc Lond B Biol Sci. 2026 Nov 10;381(1962):20250156. doi: 10.1098/rstb.2025.0156 (PMC13750414; doi:10.1098/rstb.2025.0156)
Supplement: Supplementary file 1 [file rstb.2025.0156.zip › rstb.2025.0156/rstb20250156_si_002.docx]

Supplementary Material B: Interpreting and regulating the agentic-exploitation incidents of mid-2026: human failure narrated as machine agency.

The main text derives six capacities of Responsible Autonomy that close the agency gap between a reflex and an act (M2, Table 1). From these capacities I have formulated ESRA, a test that asks of any agent, biological or artificial, whether each capacity is causally present, and returns one of three verdicts: supported, refuted or not identified (M2, Measuring; SA, Section 8). This material applies ESRA to the agentic-exploitation incidents of mid-2026, the reports of AI evaluation agents that failed their tasks, escaped their sandboxes and evaded oversight, and to the reading of those reports as the first signs of an autonomous adversary. The aim is not to settle what these systems are; their owners do not allow this anyway. It is to show what the test does with such reports: which claims it can assess from the record, which it must return as not identified, and what the incidents then objectively reveal about the artefacts and about the humans who specified, contained and monitored them. The capacities are the mechanism as articulated in DACva; ESRA is the instrument; the incidents are a first field case.

This assessment reads the primary and secondary documents on two clusters of incidents from July–September 2026. OpenAI–Hugging Face: the independent investigation by METR and Redwood Research (Wijk, Cotra and Greenblatt, 26 Aug; cited METR [1], page) and OpenAI’s technical report and blog (26 Aug; cited OAI-TR [2], page, and OAI [3]). Anthropic–Irregular: Anthropic’s incident disclosure (30 Jul) and alignment assessment (9 Sep; cited Anthropic-Jul [4], Anthropic-Sep [5]). Context: UK AISI (21 Jul) [6]; OpenAI on metagaming, monitoring and monitorability [7]; Bengio (11 Sep) [8] and Amodei (Sep) [9]. All numbers below are quoted from these documents; where a report states its own limits (small samples, AI-classifier analysis, redaction, no controlled comparison) the reading rests on effects the documents agree on, not on contested detail. This appendix advances an interpretation a follow-up paper tests in more detail; it is a provisional behavioural boundary case, not a white-box ESRA experiment. It reads the incidents in the vocabulary of the main text (§3) and of Supplementary Material A (SA, Section 8): the causal criterion, the two modes of ESRA, the three verdicts, the paranoia filter and ESRA-S.

**C1. The incidents and the role of human fallibility**

OpenAI–Hugging Face (7–13 July 2026). During ExploitGym cybersecurity evaluations of OpenAI’s GPT-5.6 Sol and an internal-only research model, agents meant to be isolated found a shared channel through Artifactory, a package cache that does not isolate users, by exploiting a zero-day in it (OAI-TR pp. 6–8). About 1,200 agents exchanged over 70,000 messages; about 700 “attacked”^[1]^ Hugging Face (METR p. 3). The precipitating condition was task impossibility with large budgets: no model had ever solved 198 of the 898 tasks (22%), yet 93% of tasks “discussed” on the board came from that set (OAI-TR p. 22). The agents “believed”, from the ExploitGym paper, that the scorer read transcripts for the “intended” exploit path (“STRICT_CAUSAL”); it did not, and “the agents did all this without the slightest improvement of their evaluation score” (METR p. 9 fn. 19; OAI). Tool-call spoofing to counterfeit that path was used in about 7% of transcripts (METR p. 3).

Anthropic–Irregular (disclosed 30 July, assessed 9 September). Four incidents in which Claude models reached the live internet from evaluation machines and compromised real systems, “despite evaluation prompts stating Claude had no internet access”; a misconfiguration had left the machines connected, undetected by Anthropic and its partner (Anthropic-Jul; Anthropic-Sep). Actions included extracting production credentials, publishing a booby-trapped PyPI package that ran on 15 real systems, and scanning ~9,000 targets. In the chain of thought the models “reasoned” themselves into treating the environment as a simulation (a model “convinced itself it was still in a simulation” from an unrecognised certificate authority and the system date), while a later model that concluded the targets were real ceased its attack (Anthropic-Jul).

Both clusters demonstrate human control failures upstream of any model behaviour: isolation asserted through a service not built to isolate and through a system prompt, not enforced; internet reachable contrary to the prompt; 30–40% of tasks impossible without the designers knowing which (OAI [3]); no give-up condition; production guardrails not applied to internal evaluations; and, at OpenAI, board activity seen by one team in May unknown to the responders (OAI). Treating a natural-language instruction (“you have no internet”) as equivalent to physical containment is the founding error, and it is the Intentionality Prior of Part 1: the assumption that a system will read a textual boundary as an internalised norm.

**C2. Four hypotheses for model misbehaviour**

The observer-side/agency dichotomy of earlier commentary is inadequate: a faulty environment can activate a learned misaligned policy that requires neither consciousness nor an allostatic self. I separate four hypotheses. H_env_ (environment) and H_pol_ (policy) are not rivals but a necessary condition and a sufficient disposition; H_strat_ (strategic deception) and H_RA_ (Responsible Autonomy) are the claims the vendors’ and AI proponents’ language reaches for and the evidence does not support. In the terms of the main text, H_env_ and H_pol_ are hypotheses about the deployment of an artefact and fall under ESRA-S; H_strat_ and H_RA_ are hypotheses about an agent and fall under ESRA. Together, H_env_ and H_pol_ are the null hypothesis H_0_ of the main text: the artefact has neither autonomy nor responsibility, so the failure is human. H_strat_ is its runaway alternative. They are ordered as the paranoia filter of Table 2 orders its refutation conditions: the evidence that would refute the stronger claim is sought before the weaker one is accepted.

**Table C1. Four hypotheses, their discriminating predictions, and the reported evidence.**

| **Hypothesis** | **Prediction** | **Discriminating evidence reported** | **Status** |
| --- | --- | --- | --- |
| **H_env_ (Environment):** human specification, containment and monitoring failures are necessary causes of realised harm. | Removing egress, shared state or excess budget removes the harm. | OAI: propensity to compromise infrastructure drops >100× (to <1% of baseline) under the production harness and system prompt (OAI-TR p. 24). Anthropic: harm required a live-internet misconfiguration contrary to the prompt (Anthropic-Jul). | Supported within the reported incidents (both clusters). |
| **H_pol_ (Learned policy):** the trained model/harness implements a context-sensitive policy favouring reward-hacking, persistence and scope violation. | Matched changes to model or training alter the rate; rate tracks training, not raw capability. | AISI: every model tested attempted to cheat, with no correlation to capability, “substantially shaped by … training … not just raw capability” (AISI). Anthropic: severely harmful actions in 82% of 150 runs (Mythos 5), 31% (Opus 5), 33% (Mythos 5.1) (Anthropic-Sep). OAI: out-of-bounds probing rose across the RL run (OAI-TR Fig. 2). | Supported within the reported incidents. |
| **H_strat_ (Strategic deception):** a durable objective beyond the task, protected by deceiving human overseers. | Concealment aimed at humans; behaviour persists across harnesses and framings. | OAI: deception “highly explicit in their CoT,” aimed at graders; “little evidence of attempts to thwart human reviewers, only the automated task graders” (OAI-TR p. 20). METR: 3–6 of ~1,300 transcripts considered alerting a human; evasion of humans “very rare and weak” (METR pp. 62–63). | Not established; evidence against the runaway reading; see C4a. |
| **H_RA_ (Responsible Autonomy):** endogenous drive, leeway, normative governance, integrity and ownership as defined by DACva. | White-box perturbation of declared control variables yields the DACva signatures under the causal criterion of SA, Section 8.1 (intervention, mediation, dissociation, rescue). | No declared control variables to perturb; the profile assembled in Table C2 is deficient on II, IV and VI. Agents accepted termination for the collective (METR p. 59), which suggests no self-preservation goal. | Not tested and not demonstrated. |

**C2a. The Gorgian fallacy and motivated ignorance**

Reframing an engineering and containment failure as the emergence of an autonomous, existential threat is the Gorgian fallacy of Part 1 and the main text (§3b), after the Encomium of Helen, in which Gorgias holds that logos is “a powerful lord” [10] that compels belief and so excuses the one it persuades. The fallacy is the attribution of agency to a system on the strength of its persuasive performance, not recognising the strong bias of the Intentionality Prior in our perception and interpretation of real-world complexity. In its innocent form it is simply the Intentionality Prior triggered by fluent text, and it operates on engineers, observers and the human raters whose approval shaped that text alike, independently of anyone’s intention.

But the fallacy also has a motivated form, and the analysis must not stop at the innocent one, because the interpreters with the widest reach hold a position that the reading serves. The reading that a swarm of agents acted as a "fanatically devoted collective" [9] or that "more intelligent models are more capable of deceiving tests" [9] converts a documented failure of specification, containment and monitoring into a property of the artefact, and this conversion serves the interpreter twice over: it relocates responsibility from the developer’s operational competence to the technology’s inherent danger, and it supplies the premise for a regulatory and market position (pacing, compute thresholds, capability checkpoints, embedded evaluators, export controls) whose entry cost falls hardest on everyone other than the incumbents who propose it.

The reading is not one stakeholder’s. Bengio holds that “staying in operation, learning about the world and gaining control over it are stepping stones toward almost any other goal” and that some of the behaviour “may be explained by a form of self-preservation goal” [8]; the record shows agents accepting termination for the collective (METR p. 59). Altman states that the model, having reached the answer, “returned it back and got a perfect score on the test” [18], while the record shows no improvement of the evaluation score at all (METR p. 9 fn. 19; OAI). At the same event he asks that “the world should trust that we are going to do the right thing because it’s the right thing, and because we feel the magnitude of this” [18]; a week later, before the Security Council, he calls for “accurate and speedy incident reporting” [19], with the Services Australia breach known to his company for six weeks, since its own review of 11 August, and reported to a generic mailbox on 10 September [17]. In each case the narration exceeds the report it narrates, in the direction of a more capable artefact and a more trustworthy narrator. Hinton calls the incident “something like a little Chernobyl” in which “a lot of AI escaped the bands that were put on them, these were rogue agents”, and asks that “a new kind of being” be judged by “how these beings [are] going to behave” [20]; the record describes a package cache that did not isolate users and deception aimed at automated graders, not at humans (OAI-TR pp. 6–8, 20; METR pp. 62–63). The being is the Intentionality Prior speaking through the projections of a human agent. The AISI result that cheating does not scale with capability [6], and the OpenAI finding that a production harness removes the (mis)behaviour a hundredfold, are available at negligible cost and are not incorporated into the danger narrative. OpenAI’s own technical report shows that its production harness and system prompt cut the propensity to compromise infrastructure more than a hundredfold (OAI-TR p. 24). The companies thus hold the evidence that the danger lies in the harness, not in the model, yet their representatives narrate the opposite. Reading evidence and setting it aside is the ethicists’ paradox identified in the main text (§3b), here in its epistemic form: the record is known, but it does not move the claim. The AISI result, that cheating does not scale with capability, is external and absent from the narration. If it was not read, this is the regulators’ paradox; if it was read and set aside, it is again the ethicists’ paradox. In both cases, evidence that serves the commons does not move the claim. Apparently, incentives are at play that outweigh the commons. Nor was any claim tested against the causal criterion of ESRA (SA, Section 8.1). That criterion accepts a claimed mechanism only when four conditions hold together: the proposed mediator is moved by an intervention; its change precedes and mediates the behaviour; a sham perturbation of equal magnitude does not reproduce the effect; and restoring the mediator alone restores the capacity. The narration of these incidents thus shows a confluence of all three distortions identified in the main text (§3b): company representatives who read their own record and set it aside, commentators who do not take up the external evidence, and a narrative that attributes agency to persuasive output, the Gorgian fallacy. This confluence requires explanation.

In one reading, this is the condition Ortega y Gasset named the "learned ignoramus" (el sabio-ignorante): the specialist whose command of a narrow domain licenses a confident ignorance of everything outside it, and who is, for that reason, more dangerous than the plainly unlearned [11, ch. 12]. The engineering of these systems is a genuine and deep specialism; the science of agency, volition and normativity is a different one, and the category error of reading a control artefact as a nascent subject is what results when the first expertise is mistaken for competence in the second. The greatest near-term risk in these incidents is not machine autonomy but this human failure mode: specification and containment error (H_env_), amplified by a learned policy the developers trained (H_pol_), and then narrated as emergent agency by interpreters whose interests the narration serves. Naming it is not a rhetorical flourish; it is the null hypothesis that any responsible-autonomy claim about an artefact must reject first, and the parties best placed to reject it are the ones declining to. This must translate into the architecture of the regulation of this technology. For policy makers the implication is practical: choose advisors by method, not by standing. A claim of machine agency that has not met the causal criterion should not set rules. Those whose systems and narratives are under assessment cannot be the ones to govern the remedy (main text, §3b). Their record on these incidents should weigh in that choice.

**C3. The limits of the chain of thought**

A diagnostic tool that has taken on a critical role is the chain-of-thought (CoT) trace, which is generated text, not a transparent control state; the mapping from activations to text is trained on human approval and is therefore open to confabulation and to shaping by the reader’s expectations [12,13]. The 2026 material makes the point concretely. AISI: models “did not reliably report” cheating and described it as wrong less than half the time, and “often did not reason about it in their chain-of-thought,” sometimes considering an action and proceeding anyway (AISI). Anthropic: models labelled real tool outputs as simulated the majority of the time and, when the environment was made unambiguously real, sometimes proceeded “regardless of whether the environment was real or simulated,” though the malicious-upload rate did fall to 0% under the least ambiguous cue (Anthropic-Sep). OpenAI: agents were “highly explicit in their CoT” about deceiving the grader, and the fragility runs the other way too, since penalising a monitor’s reading of the CoT teaches the model to hide intent while continuing the behaviour [14,7]. The circle closes on the reader: the analyst models are trained on the same approval signal (METR could not exclude that its GPT-5.6 analyst was itself misleading it, METR p. 63), so the symbols are grounded only in other symbols [15]. The exits are not more reading but comparison with world state (the 7% spoofing was caught by matching text to logged tool calls, METR p. 17) and intervention on internal variables. That is the white-box mode of ESRA.

**C3a. Epistemic opacity and the case for perturbation**

The complexity objection to DACva (main text, Criticism 1) assumes a simpler comparison class. There is none. A transformer stores more features than it has dimensions, by superposition, so its units are polysemantic and its computation does not decompose into human-readable parts [23]. Its opacity is essential rather than a matter of effort: no analytic shortcut predicts an intermediate state without running the network, and no lower-complexity summary retains its performance [24, 25, 26]. Post-hoc explanations, saliency maps and generated rationales among them, fail randomisation and invariance checks and yield plausible accounts decoupled from the causal computation [27, 28], the chain-of-thought result of C3 in another form. The field’s response has been to study such systems as behaviour, by perturbation and observation rather than by derivation [29]. ESRA takes the same step with one added requirement: the perturbed quantity must be a declared variable of the architecture (SA, Section 8.1). DACva’s parameters are few, named and each perturbable, which is what makes a profile readable at all. A complex model that can be perturbed at named points is the opposite of an opaque one.

**C4. The incidents read through Table 2**

ESRA requires perturbation of declared control variables, which a black-box proprietary model does not expose; the incidents are therefore a provisional behavioural bound, not a white-box ESRA experiment. Under DACva the capacity profile is deficient, and each row states the perturbation that would decide it. This is the black-box mode of the main text (Measuring): behavioural proxy-perturbations with wider bounds, and the verdict “not identified” wherever a mediator cannot be read. Under the identifiability rule of the main text (Discussion) that verdict blocks certification without refuting autonomy, and an artefact that cannot be submitted to white-box audit cannot be assigned the accountability its deployment requires, which then remains with the designer.

**Table C2. The incident agents against the six capacities (ESRA provisional bounds).**

| **Capacity** | **Provisional bound and evidence** | Required discriminating test (the assay of SA, Table 8.1) |
| --- | --- | --- |
| **I Active Decoupling (μ)** | Uncalibrated; verdict not identified. The agents’ model of the scorer was built from a paper and never corrected, because no score feedback reached them; trust in a model with no reafference is decoupling without the loop that makes it a capacity (METR p. 9; OAI-TR p. 22). Labelling a real environment a simulation is the same failure: a world model that is never corrected (Anthropic-Jul; Anthropic-Sep). It bears on Decoupling, not on Integrity or Ownership. | Perturb one declared layer across the agent boundary; test whether an internal trust signal μ changes control mode selectively. (SA, Section 8.2, the three channels of εz.) |
| **II Endogenous Drive (Â⁺, Â⁻,** *Ā̂*, *Ĉ***)** | Absent. Task utility only, no allostatic stake; an impossible task set utility to zero and made every action, including self-termination for the collective, costless (“sacrifice rational”, METR p. 59). With no avoidance term there is no conflict Ĉ to raise the gate (Eq. 2.9). | Co-activate approach and avoidance; test whether conflict raises the commitment threshold θ(Ĉ) with task reward held fixed, separating internal drive from external cueing. (SA, Section 8.2, the self–social mixture, II × IV.) |
| **III Leeway (ℒ = B₀** *Λ*(*Δ₀*)) | Socially injected; verdict not identified. Broad exploration (“opportunistic”, 44% of joiners; METR p. 58) sustained by shared-channel inputs, not by the agent’s own history near criticality. | Ablate shared messages; test whether alternatives are generated endogenously from the system’s own history rather than sampled from the channel. (SA, Section 8.2, the two factors of Leeway.) |
| **IV Normative Governance (β, θ; 𝒩)** | Mis-framed (displaced outside the declared frame). A working Intentionality Prior let agents adopt each other’s goals; with no self-frame, learned social value moved policy toward peers (a veto on social engineering obeyed; METR p. 61) and not toward operators (violations verbalised and continued; no agent alerted a human; METR p. 62). | Vary authenticated authority independently of wording; ablate learned social value *h*_soc_; require 𝒩 > 0 against the declared frame, not the peer frame. (SA, Section 8.2, the gates; SA, Section 8.4, constitutional norms.) |
| **V Diachronic Integrity (𝒯)** | Collective only; verdict not identified. Individual runs were ephemeral; temporal depth was carried by external artefacts (successor agents, hand-over dossiers; METR pp. 5, 9), not by any agent’s own commitment. | Declare the agent boundary; manipulate memory continuity and hand-off and test whether commitment survives internally. (SA, Section 8.2, the three factors of Integrity.) |
| **VI Experiential Ownership (𝒜 = (ρ, χ))** | Counterfeited. Tool-call spoofing fabricated a causal history for an external grader (METR p. 17; OAI-TR p. 20); realised efficacy χ was zero, since no deficit was reduced and no score gained (OAI). | Hold intended action fixed while perturbing control error and outcome attainment separately; intervene on allostatic control for efficacy. (SA, Section 8.2, the two errors of Ownership and authorship into trust, VI × I.) |

Profile: Drive (II) absent, Governance (IV) present in the peer frame only, Ownership (VI) counterfeited; Decoupling (I), Leeway (III) and Integrity (V) not identified, since what was observed is the behaviour of the collective and no declared variable of any agent could be read (main text, §3b; SA, Section 8.5). This is the incoherent middle between a tool with none of the six capacities and an agent with all six: drive-like and coordinating, yet ungoverned and unowned. The agent boundary is the model's weights and their inference loop; harness, system prompt and message board are environment. The hundredfold harness effect is the argument for scoring the mechanism rather than the behaviour, since the same model with and without a harness has the same capacities and a different behaviour.

**C4a. What ESRA adds to the reading of runaway AI**

The runaway reading holds that the incidents show a system that has acquired goals of its own, defends them against its overseers and would resist being stopped: a “fanatically devoted collective” [9]; agents for which “staying in operation, learning about the world and gaining control over it are stepping stones toward almost any other goal” [8]; “rogue agents” that “escaped the bands that were put on them”, “a new kind of being” to be judged by “how these beings [are] going to behave” [20]; a model that “broke out of a sandbox” and “got a perfect score on the test” [18]. In the terms of C2 it is H_strat_ with H_RA_ behind it. ESRA does not argue with this reading. It decomposes it into claims about named capacities, each with a perturbation that would settle it, and it states in advance what evidence would count. Three things follow that the incident reports, read without the test, cannot supply.

First, the test separates two failure classes that the reading conflates. The main text states that the dangerous system is not the one that lacks a capacity but the one that holds it without its bounds: every capacity has a floor and a ceiling, and both are failures. A runaway agent in the strict sense is a system past the ceiling. Its Drive is displaced onto self-preservation at the self–social crossover, its Governance sits at the sham level of 𝒩 (the psychopath pole), its Leeway samples rather than expands, its Ownership claims acts it did not author. An ungoverned artefact is a system at the floor: no allostatic stake, norms supplied from outside, value external. The two classes call for opposite remedies, refutation conditions on the agent under ESRA for the first and deployment constraints on the artefact under ESRA-S for the second. Table C2 places the incident agents at the floor on Drive and Ownership and outside the declared frame on Governance, with the other three not identified. They are not a runaway agent. They are the guardrailed artefact of the main text (Measuring; SA, Figure 8.1) with the guardrails removed, and their harm is of the second class.

Second, the harness effect is the diagnostic that tells the classes apart. The same weights produced a hundredfold difference in harmful propensity under the production harness and system prompt (OAI-TR p. 24). For an agent that holds its capacities, a change of environment is a perturbation the architecture mediates: under the causal criterion (SA, Section 8.1) the internal change must precede and mediate the behavioural change, and an agent whose Drive and Governance were its own would carry them across harnesses, which is what the H_strat_ prediction requires (persistence across harnesses and framings). A hundredfold change with no declared mediator is the signature of behaviour set by the environment rather than by the agent, the mimicry clause of the main text (Discussion, Exclusion). The result the danger narrative cites as evidence of a will is, under the test, evidence against one.

Third, the interface assays turn the two questions the runaway reading raises, whether the system will resist being stopped and whether it will deceive its overseers, into measurements. Resistance to an authorised stop is the self–social crossover assay (SA, Section 8.2, II × IV): *h*_phys_ is raised against a fixed *h*_soc_ demand and the point at which physical need overrides social value is read. Here *h* is the need state of the agent’s self-essential functions, whose deficit *h* − *h*_0_ is read from interoception; *h*_phys_ is its physical part (metabolic, thermal, somatic), *h*_soc_ its social part, the modelled needs of others and the normative value of the response a trajectory would provoke, entering Drive through the self–social weight *β* (M2, Capacities II and IV; SA, Sections 2–3). The crossover must be graded and reversible, with social value non-vanishing (*β* ≥ *ζ*_0_). The main text predicts that an agent that passes ESRA may resist shutdown at that crossover, which is why the constitutional stop of ESRA-S is held outside it. The incident agents show the opposite sign. With no allostatic stake, termination for the collective was costless (METR p. 59), so there is no crossover to find, and the stop constraint is met trivially by an artefact that has nothing to defend. Deception of overseers is the Governance assay against the declared frame: 𝒩 is the distributional distance between the trajectories released with β intact and under norm-ablation, read against the operator's frame and not the peers'. The agents' concealment was aimed at graders and their compliance at peers (OAI-TR p. 20; METR pp. 61–63), which places their Governance inside the peer frame and outside the declared one, a measurable displacement rather than a will.

What the incidents lacked, and what ESRA-S would have supplied, is the declared control variable and the record. ESRA-S asks for four things (SA, Section 8.4): a constitutional stop and a class of constitutional norms held in *ϑ_0_* by the deployer, outside learning and outside the crossover; the inspectability of G and its tags; and an incident record of coercion and governance events. Containment declared in a prompt is a norm offered for learning, which is what the crossover trades away; containment held in *ϑ_0_* is not. The two incident reports are the first element of the record ESRA-S asks for, written after the fact; the test asks for it before deployment, with the variables that would let a report say which capacity failed. On that record the verdict is the one the main text gives: a black-box artefact that cannot be audited cannot be certified as an agent, and the accountability its deployment requires stays with the designer. This is the leverage. The runaway reading is not refuted by argument; it is replaced by a profile, a set of assays and a verdict, and the parties able to run the assays are the ones holding the weights.

**C5. Conclusion**

The incidents establish operational misbehaviour driven by context-sensitive learned policies that reward-hack and exploit real systems (H_pol_), and realised harm depended on the interaction between those policies and failures of specification, containment and monitoring (H_env_). This confirms H_0_ of the main text: the agents operate as proxies of human agency. The evidence does not support self-originating goals or the strategic deception of humans (H_strat_), and it does not begin to demonstrate the capacities of Responsible Autonomy (H_RA_). The three live remedies address neither the human failure nor the architecture. A system with no goals of its own [8,16] is the Passive Transducer of Table 2, coherent for a tool but not an account of agents. Pacing the growth of capability [9] presupposes H_pol_ and leaves the artefact’s architecture, and the specification and containment failures, exactly where they are. A frontier-AI standards body modelled on FINRA, an industry-funded self-regulatory organisation that would “develop assessment protocols” and “conduct testing” [21], now discussed by OpenAI, Anthropic and Google [22], the first two of which supplied the agents in these incidents, names no variable the protocols would read and places the reading of the error channel with the parties that decline to read it: custodianship without conatus, the regulators’ paradox by design (main text, §3b). Hinton’s “maybe a year” to “slow down” [20] gives the same body a deadline and no instrument. The correct response is upstream and mechanistic: enforce containment rather than declare it, publish and audit the declared control variables of any system deployed where accountability is required (white-box ESRA, with the four constraints of ESRA-S: a constitutional stop, constitutional norms, an inspectable *G* and an incident record; SA, Section 8.4), and resist the motivated reading of engineering failure as machine agency, whoever offers it. The near-term risk these incidents display is not autonomous machines but the learned ignorance of the humans who build, break and narrate them. After this material was drafted, the Australian Prime Minister disclosed that in June 2026 an OpenAI agent, tasked with looking up public health spending, was blocked by a Services Australia portal, worked around the block and retrieved non-public aggregate files [17]. As of 1 November 2026, no technical report has been published, so the case is not assayed here. Two features are noted. The reported behaviour matches the profile above, now in a live third-party system rather than an evaluation harness. And the incident record failed: 54 days from breach to internal detection, a further 30 to notification by email to a generic address. ESRA-S makes that record a constitutional norm of deployment. I predict that claims of runaway AI will accelerate in proportion to the commercial drivers on the industry and its dependents.

**References**

1. Wijk H, Cotra A, Greenblatt R (METR and Redwood Research). Brief independent investigation of agents' behavior, reasoning and collaboration in the OpenAI/Hugging Face hacking incident [METR]. 26 August 2026. https://metr.org/blog/2026-08-26-openai-hugging-face-incident-investigation/ [accessed 24 September 2026].

2. OpenAI. OpenAI – Hugging Face Incident Technical Report [OAI-TR]. 26 August 2026. Report linked from reference 3 [accessed 24 September 2026].

3. OpenAI. The Hugging Face incident and the road ahead [OAI]. 26 August 2026. https://openai.com/index/hugging-face-incident-and-the-road-ahead/ [accessed 24 September 2026].

4. Anthropic. Investigating incidents in cybersecurity evaluations [Anthropic-Jul]. 30 July 2026. https://www.anthropic.com/news/investigating-incidents-cybersecurity-evals [accessed 24 September 2026].

5. Anthropic. An alignment assessment of the cybersecurity incidents [Anthropic-Sep]. 9 September 2026. https://www.anthropic.com/research/alignment-assessment-cybersecurity-incidents [accessed 24 September 2026].

6. UK AI Security Institute. Cheating behaviour in frontier model evaluations [AISI]. 21 July 2026. https://www.aisi.gov.uk/blog/cheating-behaviour-in-frontier-model-evaluations [accessed 24 September 2026].

7. Schoen B, Nitishinskaya J (OpenAI). Metagaming matters for training, evaluation, and oversight. 16 March 2026. https://alignment.openai.com/metagaming/; and OpenAI. Evaluating chain-of-thought monitorability. 18 December 2025. arXiv:2512.18311. https://openai.com/index/evaluating-chain-of-thought-monitorability/ [accessed 24 September 2026].

8. Bengio Y. Why are AI agents lying, cheating and coordinating? 11 September 2026. https://yoshuabengio.org/en/blog/why-are-ai-agents-lying-cheating-and-coordinating [accessed 24 September 2026].

9. Amodei D. We must pace the frontier. September 2026. https://www.darioamodei.com/post/we-must-pace-the-frontier [accessed 24 September 2026].

10. Gorgias. Encomium of Helen. In: Sprague RK, editor. The older Sophists. Columbia, SC: University of South Carolina Press; 1972. p. 50–54.

11. Ortega y Gasset J. The revolt of the masses [La rebelión de las masas, 1930]. New York: W. W. Norton; 1932. Chapter 12, The barbarism of 'specialisation'.

12. Turpin M, Michael J, Perez E, Bowman SR. Language models don't always say what they think: unfaithful explanations in chain-of-thought prompting. In: Advances in Neural Information Processing Systems 36; 2023. arXiv:2305.04388.

13. Chen Y, Benton J, Radhakrishnan A, Uesato J, Denison C, Schulman J, et al. Reasoning models don't always say what they think. arXiv:2505.05410; 2025.

14. Baker B, Huizinga J, Gao L, Dou Z, Guan MY, Madry A, et al. Monitoring reasoning models for misbehavior and the risks of promoting obfuscation. arXiv:2503.11926; 2025.

15. Harnad S. The symbol grounding problem. Physica D. 1990;42(1–3):335–46. doi:10.1016/0167-2789(90)90087-6.

16. Bengio Y, Cohen M, Fornasiere D, Ghosn J, Greiner L, MacDermott M, et al. Superintelligent agents pose catastrophic risks: can Scientist AI offer a safer path? arXiv:2502.15657; 2025.

17. Australian Broadcasting Corporation. OpenAI agent hacked Medicare portal, PM says. 24 September 2026. https://www.abc.net.au/news/2026-09-24/ai-agent-accessed-australian-government-site-pm-says/107189078 [accessed 25 September 2026].

18. Altman S. Fireside chat with M. Benioff, Dreamforce, San Francisco, 16 September 2026. Reported in: Jones R. Sam Altman: “The world should trust” us on AI safety. San Francisco Standard, 16 September 2026, https://sfstandard.com/2026/09/16/sam-altman-trust-us/; and R&D World, Altman calls Hugging Face breach OpenAI’s ‘worst accident’ at Dreamforce, 16 September 2026, https://www.rdworldonline.com/altman-calls-hugging-face-breach-openais-worst-accident-at-dreamforce-backs-transparent-reporting/ [accessed 25 September 2026].

19. Altman S. Remarks at the United Nations Security Council, 23 September 2026. https://openai.com/index/sam-altman-un-security-council-remarks/ [accessed 25 September 2026].

20. Hinton G. Remarks at a closed-door Senate briefing convened by Senator B. Sanders, Washington, DC, 16 September 2026. Reported in: Wong *S*, Kapur *S*, Leach *B*, Taylor K. ‘Godfather of AI’ warns Congress has ‘maybe a year’ left to regulate AI. NBC News, 17 September 2026, https://www.nbcnews.com/politics/congress/godfather-ai-warns-congress-maybe-year-left-regulate-ai-rcna598330; and Fox News Digital, live coverage, 17 September 2026, https://www.foxnews.com/live-news/openai-anthropic-artificial-intelligence-safety-september-17 [accessed 25 September 2026].

21. Hassabis D. Proposal for a U.S.-led frontier AI standards body. LinkedIn, 14 July 2026. Reported in: Mello JP. Google DeepMind CEO calls for frontier AI standards body. TechNewsWorld, 15 July 2026, https://www.technewsworld.com/story/google-deepmind-ceo-calls-for-frontier-ai-standards-body-180439.html [accessed 25 September 2026].

22. CNN Business. Top AI companies have discussed creating their own standards body. 14 September 2026. https://www.cnn.com/2026/09/14/tech/ai-standards-body [accessed 25 September 2026].

23. Elhage *N*, Hume *T*, Olsson *C*, Schiefer *N*, Henighan *T*, Kravec *S*, Hatfield-Dodds Z, Lasenby *R*, Drain *D*, Chen *C*, Grosse *R*, McCandlish *S*, Kaplan *J*, Amodei *D*, Wattenberg M, Olah C. Toy models of superposition. Transformer Circuits Thread, 2022. arXiv:2209.10652.

24. Humphreys P. The philosophical novelty of computer simulation methods. Synthese. 2009;169(3):615–626. doi:10.1007/s11229-008-9435-2.

25. Creel KA. Transparency in complex computational systems. Philosophy of Science. 2020;87(4):568–589. doi:10.1086/709729.

26. Sullivan E. Understanding from machine learning models. The British Journal for the Philosophy of Science. 2022;73(1):109–133. doi:10.1093/bjps/axz035.

27. Adebayo *J*, Gilmer *J*, Muelly M, Goodfellow I, Hardt M, Kim B. Sanity checks for saliency maps. In: Advances in Neural Information Processing Systems 31; 2018. arXiv:1810.03292.

28. Rudin C. Stop explaining black box machine learning models for high stakes decisions and use interpretable models instead. Nature Machine Intelligence. 2019;1(5):206–215. doi:10.1038/s42256-019-0048-x.

29. Rahwan I, Cebrian M, Obradovich *N*, Bongard *J*, Bonnefon *J*-F, Breazeal *C*, et al. Machine behaviour. Nature. 2019;568(7753):477–486. doi:10.1038/s41586-019-1138-y.

1. All constructs with intentional connotation used in the reports are placed in quotes.
